# Responses of DNA Mismatch Repair Proteins to a Stable G-Quadruplex Embedded into a DNA Duplex Structure

**DOI:** 10.3390/ijms21228773

**Published:** 2020-11-20

**Authors:** Anzhela V. Pavlova, Mayya V. Monakhova, Anna M. Ogloblina, Natalia A. Andreeva, Gennady Yu. Laptev, Vladimir I. Polshakov, Elizaveta S. Gromova, Maria I. Zvereva, Marianna G. Yakubovskaya, Tatiana S. Oretskaya, Elena A. Kubareva, Nina G. Dolinnaya

**Affiliations:** 1 Department of Chemistry, Lomonosov Moscow State University, Leninskie Gory 1, 119991 Moscow, Russia; natasha298@mail.ru (N.A.A.); gennadylaptev@gmail.com (G.Y.L.); gromova@belozersky.msu.ru (E.S.G.); zvereva@genebee.msu.ru (M.I.Z.); dolinnaya@hotmail.com (N.G.D.); 2 Belozersky Institute of Physico-Chemical Biology, Lomonosov Moscow State University, Leninskie Gory 1, 119991 Moscow, Russia; monakhovamv@gmail.com (M.V.M.); oretskaya@belozersky.msu.ru (T.S.O.); kubareva@belozersky.msu.ru (E.A.K.); 3 Institute of Carcinogenesis, N.N. Blokhin NMRCO, Kashirskoe Shosse 24, 115478 Moscow, Russia; ann.ogloblina@gmail.com (A.M.O.); mgyakubovskaya@mail.ru (M.G.Y.); 4 Faculty of Fundamental Medicine, Lomonosov Moscow State University, Lomonosovsky Avenue 27/1, 119991 Moscow, Russia; vpolsha@mail.ru

**Keywords:** G-quadruplex, DNA mismatch repair, MutS, MutL, MutH, protein–DNA binding

## Abstract

DNA mismatch repair (MMR) plays a crucial role in the maintenance of genomic stability. The main MMR protein, MutS, was recently shown to recognize the G-quadruplex (G4) DNA structures, which, along with regulatory functions, have a negative impact on genome integrity. Here, we studied the effect of G4 on the DNA-binding activity of MutS from *Rhodobacter sphaeroides* (methyl-independent MMR) in comparison with MutS from *Escherichia coli* (methyl-directed MMR) and evaluated the influence of a G4 on the functioning of other proteins involved in the initial steps of MMR. For this purpose, a new DNA construct was designed containing a biologically relevant intramolecular stable G4 structure flanked by double-stranded regions with the set of DNA sites required for MMR initiation. The secondary structure of this model was examined using NMR spectroscopy, chemical probing, fluorescent indicators, circular dichroism, and UV spectroscopy. The results unambiguously showed that the d(GGGT)_4_ motif, when embedded in a double-stranded context, adopts a G4 structure of a parallel topology. Despite strong binding affinities of MutS and MutL for a G4, the latter is not recognized by *E. coli* MMR as a signal for repair, but does not prevent MMR processing when a G4 and G/T mismatch are in close proximity.

## 1. Introduction

G-quadruplexes constitute one of the most amazing and extensively studied noncanonical forms of DNA; their presence throughout the genome is rigorously proved in vivo [1,2]. Endogenous G4s are formed by intramolecular interactions of DNA sequences containing G-rich tracts through the stacking of coplanar arrangements of four guanines, i.e., G-quartets, which are stabilized by Hoogsteen hydrogen bonds and by interactions with metal ions that are coordinated in the central cavity. G4-forming sequences (G4-motifs) are known to be highly abundant in the genomic DNA of eukaryotes, particularly in DNA telomeres and the proximal promoter region of genes, mainly oncogenes and genes involved in growth control [3,4,5].

Being structural elements of the genome, G4s are recognized by numerous cellular proteins and enzymes and interfere with very basic biological processes such as DNA replication, chromosome end protection, transcription, mutagenesis, and DNA recombination by performing regulatory functions [6,7,8,9]. On the other hand, G4 formation can promote genome instability, implicating G4s in disease and evolution. These unusual DNA structures constitute an obstacle to replication machinery, and defects in the repair systems that contribute to G4 resolution can stall a replication fork, thereby giving rise to double-strand breaks and genetic changes (e.g., inversions, recombination, mutations, and deletions) associated with cancer and neurological disorders [10].

Given the negative impact of G4s on genome integrity, it is of prime interest to understand the effects of a quadruplex on repair machinery. Among identified G4-binding proteins, there are many G4-resolving and repair proteins, such as special helicases and proteins involved in homologous recombination, base excision repair (BER), or nucleotide excision repair (NER) [11,12].

Herein, we focused on studying the impact of G4s on the mismatch repair pathway, which plays a central role in the maintenance of genomic stability. MMR, which requires precise cooperation of its constituents, improves the fidelity of DNA replication by up to 3 orders of magnitude. At the same time, the MMR system has also been reported to be a driver of certain mutations, including disease-associated trinucleotide repeat instability in human cells [13]. Importantly, the main features of MMR systems have been conserved throughout evolution from bacteria to humans [14]. Some noncanonical DNA structures can lead to disruption of MMR function and, as a consequence, to an increase in cell survival associated with carcinogenesis in eukaryotes. Likewise, defects in the mismatch repair system of prokaryotes elevate mutagenesis rate and interspecies gene transfer, which ensures the adaptability of bacteria to stress conditions and the action of antibiotics. Thus, understanding the role of G4s in MMR initiation is essential and relevant for both basic research and medical applications.

The most studied and widely employed MMR systems are those of *E. coli* and humans. In *E. coli*, the repair process is initiated by the binding of the MutS protein to mismatched or unpaired nucleotides that escaped the built-in proofreading activity of DNA replication machinery. After mismatch recognition, MutS recruits MutL in an ATP-dependent manner to form a ternary complex that is believed to coordinate a cascade of subsequent events. MutL stimulates the MutH endonuclease, which interprets the absence of DNA methylation as a mark of a daughter strand, thus assisting in the discrimination and nicking of a newly synthesized strand (methyl-directed MMR). In eukaryotes and most bacteria, MutL rather than MutH has the endonuclease activity (methyl-independent MMR). Then, a nonmethylated DNA strand is hydrolyzed by a set of exonucleases. Finally, DNA polymerase and ligase fill the resulting gap in the daughter strand (for a review, see reference [15]). Among all MMR proteins, only the binding of the main protein taking part in the initial stage of the MMR pathway, MutS, to a G4, has been studied. MutS operates in many prokaryotic and eukaryotic species. In addition to recognizing mismatches and insertion–deletion loops, *E. coli* MutS (ecMutS) and eukaryotic MutS homologs have an extraordinary capacity to recognize unrelated types of DNA lesions, including base modifications (UV photoproducts, 8-oxoguanine, and cisplatin crosslinks) and various types of non-B form structures such as DNA loops, bubbles, hairpins, and Holliday junctions [16,17,18]. To our knowledge, there are only two papers in which the binding of ecMutS and human homolog MutSα to a G4 has been reported [19,20]. EcMutS was shown to specifically recognize intermolecular polymorphic G4 DNA with affinity stronger than that for the G/T mismatch. Nonetheless, the modes of MutS binding to a G4 and to a mismatched base pair turned out to be different: (i) the highly conserved phenylalanine residue in MutS’s Phe-X-Glu structural motif, critical for G/T mismatch recognition because of stacking with one of the mispaired bases, is not required for G4 recognition; (ii) ATP-induced MutS conformational changes that promote the release of the mismatch-containing DNA duplex contrast with ATP-independent binding of MutS to a G4 structure [19].

In this study, we propose a conceptually new approach to clarify the role of G4 recognition by the MutS protein in a cascade of subsequent events and in the general regulation of MMR pathways in two bacterial species: the γ-proteobacterium *E. coli* and α-proteobacterium *R. sphaeroides.* It should be noted that G4 sequence motifs have been well documented in all bacterial genomes available in the NCBI database, although their functions are not fully elucidated [21]. The *E. coli* MMR system was chosen here as a comparison standard associated with prior research data. *E. coli* has been reported to be useful as a model microorganism for functional studies of DNA repair [22]. In addition, it was *E. coli* where the formation of G4 structures was detected in vivo [23]. As for *R. sphaeroides*, it is a highly adaptive purple bacterium that can survive in various environmental conditions. This property of *R. sphaeroides* probably has something to do with the potent functioning of repair systems including methyl-independent MMR [24].

The main aim of our study was to answer the following questions: whether G4 formation interferes with mismatch-provoked incision of DNA caused by the coordinated actions of proteins MutS, MutL, and MutH, and whether the G4 itself activates MMR responses. To this end, in addition to ecMutS, we first studied the binding affinities of G4s for other proteins involved in the initial steps of MMR: proteins MutL and MutH from *E. coli* (ecMutL and ecMutH, correspondingly) and MutS from *R. sphaeroides* (rsMutS) as well as assessed the impact of a G4 on the functioning of *E. coli* MMR.

**Figure 1:**
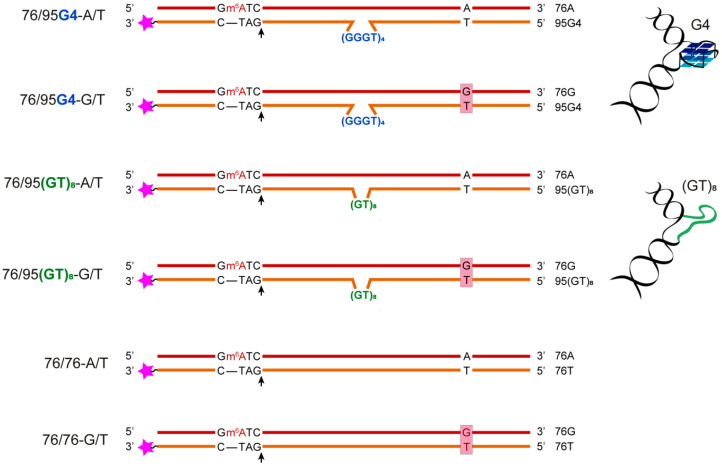
The DNA models applied to study the interactions of a G4 with MMR proteins. The DNA sequences are presented in the Supplementary Materials. The names of DNA duplexes are shown on the left, and the names of the single-stranded oligonucleotides are presented on the right. The 5′-Gm^6^ATC-3′/3′-CTAG-5′ sequence corresponds to a site recognized by MutH, where m^6^A stands for N^6^-methyl-2’-deoxyadenosine, and black arrows indicate the position of DNA hydrolysis by the MutH endonuclease. Pink asterisks represent the 5-carboxytetramethylrhodamine (TAMRA) fluorophore for DNA labeling.

To monitor the G4-mediated responses of MMR proteins, we designed and synthesized previously unreported linear DNA constructs containing biologically relevant intramolecular G4 DNA flanked by double-stranded regions upstream and downstream of the G4 structure along with the entire set of features necessary for the initiation of MMR (a G/T mismatch and MutH recognition site). The secondary structure of the engineered DNA models as well as G4 folding topology and thermal stability of both the G4 and duplex domains (Figure 1) were analyzed by a variety of biophysical and biochemical techniques: NMR spectroscopy, chemical probing, a new fluorescence method, circular dichroism (CD) analysis, and UV spectroscopy. Then, DNA models containing a G4 together with the MMR functional elements were used to assess binding affinities and the functional responses of key proteins participating in the initial stage of MMR from different bacterial sources: *R. sphaeroides* and *E. coli*.

## 2. Results

### 2.1. Design of the DNA Models

With the aim to clarify whether G4s can influence the main properties of proteins involved in the initial stage of MMR in two species of bacteria with different MMR mechanisms, we created DNA models containing an intramolecular G4 stabilized in a double-stranded DNA context. As a basic quadruplex-forming motif, we chose the d(GGGT)_4_ sequence, known for its strong ability to fold into a G4 with parallel topology and for usefulness in G4-based molecular assays.

With the exception of the single-stranded telomeric DNA, all genomic G-rich sequences are always present along with their C-rich complements, and G4 formation competes with the corresponding Watson–Crick duplex. G4 formation, therefore, first requires local dissociation of duplex strands. Several in vitro studies have shown that in linear fragments under physiological conditions, G-rich sequences usually form DNA duplexes rather than G4s [25,26]. To preclude this outcome, we designed a stable analog of a G4 within a double-helical context. The newly developed DNA model was generated by hybridization of partly complementary strands, one of which contained the G4 motif d(TT-(GGGT)_4_-T) flanked with oligonucleotide fragments, while the opposite strand lacked the site complementary to the G4-forming insert. Therefore, in contrast to surrounding sequences, the central moiety cannot flip into B-DNA and compete with G4 formation. Steric factors determined by quadruplex topology greatly affect the ability of a G4 to coexist with a nearby duplex structure in the same DNA molecule. While antiparallel G4s have ends on the same side as their quadruplex core, the parallel type has ends on the opposite side, thereby facilitating its own placement within a double helix without any structured linker, unlike the antiparallel G4 [27]. The main working DNA models, 76/95 and 76/76, prepared by direct oligonucleotide synthesis, differ in the presence of a central insert (d(GGGT)_4_ or the control d(GT)_8_ sequence of the same size, which is unable to form a stable G4 structure within the looped area [28] and the presence of functional sites for MMR initiation: MutH recognition site 5′-Gm^6^ATC-3′/3′-CTAG-5′ (m^6^A is N^6^-methyl-2’-deoxyadenosine), the G/T mismatch, or a canonical A/T base pair (Figure 1). It should be noted that the flanking regions do not contain the sequences complementary to d(GGGT)_4_ and cannot interfere with the G4 folding.

Single-stranded 95-nt DNAs with corresponding inserts were used to compare the potential for G4 formation in single-stranded and duplex surroundings. Duplex models 76/76 without a looped area containing either A/T or G/T base pairs at suitable positions were formed by hybridization of two complementary 76-nt strands (Figure 1).

### 2.2. Monitoring of G4 and Duplex Structures within the Long DNA Constructs by ^1^H NMR Spectroscopy

The secondary structures formed by 76/95 DNA models with d(GGGT)_4_ or d(GT)_8_ inserts as well as related DNA models of different composition and length (Figure 1) were first evaluated by ^1^H NMR spectroscopy. Exchangeable imino protons of the guanines in the G-quartet observed in H_2_O have characteristic chemical shifts in the region between 10 and 12 ppm [29]. This region stands apart from the chemical shift region (12–14 ppm) characteristic of imino protons taking part in regular Watson–Crick base pairing [30]. Thus, the detection of ^1^H imino resonances definitely indicates the formation of a G4 and/or DNA duplex domains in the designed DNA models.

**Figure 2:**
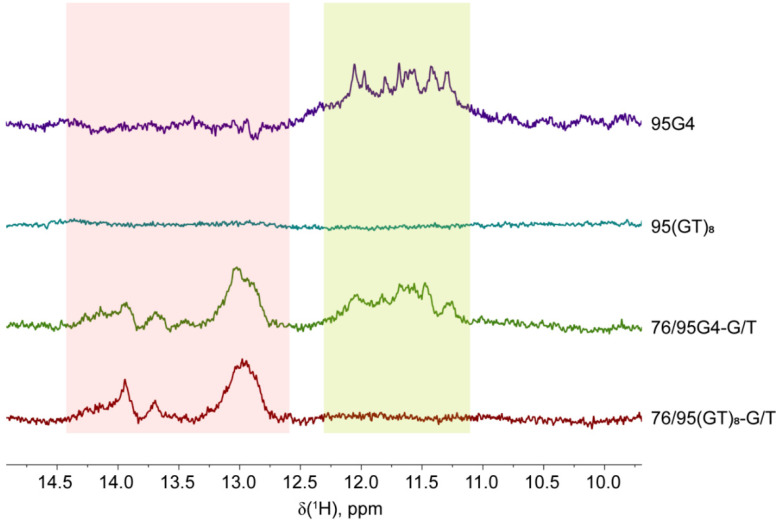
^1^H NMR spectra of single- and double-stranded DNAs. The peaks belonging to the exchangeable imino protons of the guanines involved in G-quartet formation are highlighted in yellow. The region characteristic of imino protons participating in Watson–Crick base pairing is highlighted in pink. The spectra were acquired on a 600 MHz spectrometer at 25 °C in 10 mM potassium phosphate buffer (pH 8.0) supplemented with 50 mM KCl. DNA concentrations were ~0.2 mM.

The 1D ^1^H NMR spectra of all analyzed DNA models with long stretches of potential duplex-forming sequences (76/76-G/T, 76/95 (GT)_8_-G/T, and 76/95G4-G/T) showed multiple overlaying signals of low intensity in the imino region from 13 to 14 ppm, denoting the formation of double-helical structures (Figure 2). No signals were detectable in the 10–12 ppm imino region of ^1^H NMR spectra of single- or double-stranded DNAs with no insert or with the d(GT)_8_ insert, 76/76-G/T, 95(GT)_8_, and 76/95(GT)_8_-G/T, indicating the absence of a G4 structure. At the same time, partially resolved signals in the region from 11 to 12 ppm, characteristic of Hoogsteen hydrogen bonds, were identified in the potassium ion-containing solutions of single-stranded and double-stranded 95G4 and 76/95G4-G/T samples, respectively, suggesting G4 formation by the d(GGGT)_4_ insert (Figure 2). Despite the obvious similarity, the imino proton resonances (11–12 ppm) in the NMR spectrum of 95G4 were found to be better resolved than those in the 76/95G4-G/T spectrum, probably owing to the lower molecular weight of single-stranded DNA. From the obtained NMR data, we can conclude that the d(GGGT)_4_ insert adopts similar G4 conformations in single- and double-stranded DNA contexts, although the broad and overlapping imino proton signals of guanines hinder a detailed structural analysis.

The ^1^H NMR analysis was not extended to double-stranded DNA models containing a monomethylated m^6^A site in the duplex region because this modification, at a distance from the d(GGGT)_4_ insert, should not affect G4 formation.

### 2.3. Chemical Probing Assays Clearly Indicate the Existence of G4 and Duplex Structures in 76/95 DNA Models

In an attempt to independently identify the structural potential of our DNA models, two types of chemical probing (with dimethylsulfate (DMS) or KMnO_4_) were applied. DMS most effectively methylates N7 of guanines, thereby leading to facile depurination and strand cleavage after subsequent treatment with piperidine. This nitrogen is occluded in G-quartets, and DMS protection has therefore been widely utilized to assess G4 formation [31,32,33,34]. Potassium permanganate in combination with tetraethylammonium chloride was found to oxidize all mismatched or bulged T residues (including those in G4 loops) to thymine glycol by an out-of-plane attack on the 5,6 double bond and to induce DNA cleavage at these DNA sites. In contrast, thymine residues involved in Watson–Crick base pairing are not the targets of the KMnO_4_ attack [33,35,36].

**Figure 3:**
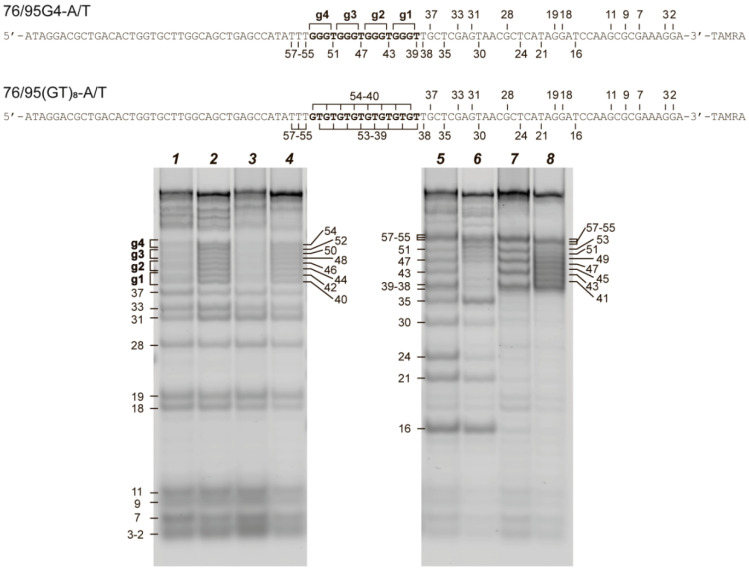
Chemical cleavage products of single- and double-stranded DNA models. A comparison of DMS (left panel) and KMnO_4_ (right panel) cleavage patterns of oligonucleotides 95G4 (lanes *1*, *5*) and 95(GT)_8_ (lanes *2*, *6*) as well as DNA duplexes 76/95G4-A/T (lanes *3*, *7*) and 76/95(GT)_8_-A/T (lanes *4*, *8*) labeled at the 3′ ends with the TAMRA fluorophore. The locations of G_3_ tracts are indicated by the brackets on the left (left panel). The numbering of nucleotide units is indicated in sequences from 3′ to 5′: left to right (two sequences at the top) and on left and right sides of electropherograms.

For chemical probing assays, we used 3′-TAMRA-labeled single-stranded 95G4 and 95(GT)_8_ DNAs containing d(GGGT)_4_ and d(GT)_8_ inserts, respectively, as well as their hybridization products with a partially complementary 76-nt DNA: 76/95G4-A/T and 76/95(GT)_8_-A/T. These experiments were conducted in the presence of potassium ions (100 mM KCl) to facilitate G4 formation. The cleavage products of the selected DNA samples after treatment with DMS or KMnO_4_ were visualized by denaturing gel electrophoresis (Figure 3). As depicted in Figure 3, the guanine residues from the d(GGGT)_4_ insert in 95G4 and 76/95G4-A/T samples have clearly lower reactivity toward DMS as compared with other guanines in the rest of the sequences (Figure 3, left panel). These data provide good evidence for the involvement of guanines from the d(GGGT)_4_ insert in Hoogsteen base pairing and indicate successful G4 formation in single- and double-stranded contexts. In contrast, each guanine in the GT repeats proved to have the same DMS reactivity as did the guanine residues in the surrounding sequences in the single- and double-stranded DNA samples. These patterns suggest that the d(GT)_8_ insert does not form a G4 structure.

The right panel in Figure 3 shows the reaction of KMnO_4_ with the same DNA samples. The number and length of the cleavage products formed after treatment of 95G4 and 76/95G4-A/T with KMnO_4_ indicate the sensitivity of thymine residues corresponding to G4 loops and of the unpaired TT residues adjacent to the d(GGGT)_4_ insert to this reagent. These findings provide additional evidence for G4 folding. Meanwhile, the attenuation of the bands corresponding to double-stranded regions directly confirms the existence of duplex flanks in the 76/95G4-A/T and 76/95(GT)_8_-A/T samples (Figure 3, right panel).

### 2.4. CD and UV Spectroscopy Confirm the Existence of a Parallel G4 Structure Folded within the DNA Double Helix

**Figure 4:**
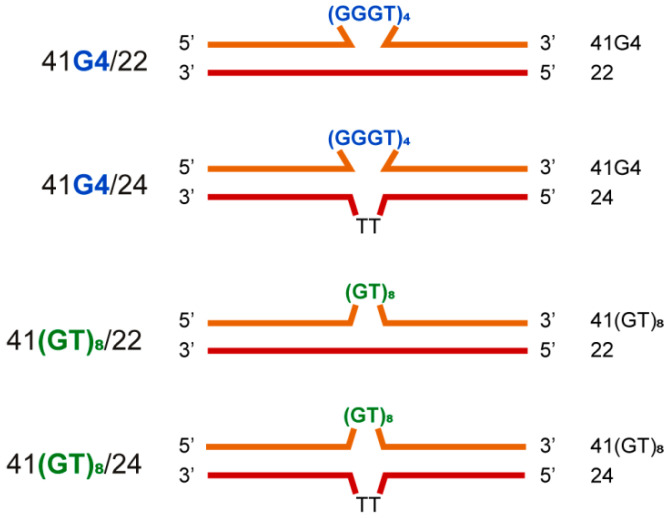
DNA models used for spectroscopic studies. DNA sequences are presented in the Supplementary Materials. The names of DNA duplexes are shown on the left, and the names of the single-stranded oligonucleotides are given on the right.

We applied a combination of CD and UV spectroscopy to further characterize the designed models. CD makes it possible to determine G4 topology [37], whereas UV spectroscopy allows for independent monitoring of G4 and DNA duplex unfolding [38]. For optical measurements, the shorter double-stranded DNA models (Figure 4) and their single-stranded components were used to improve the signal-to-noise ratio, because G4-flanking sequences that contribute to CD and UV spectroscopy signals do not participate in the folding/unfolding of G4. Together with the 41/22 models (41G4/22 and 41(GT)_8_/22), we examined 41/24 ones (41G4/24 and 41(GT)_8_/24), in which partially complementary shorter strands included two additional T residues in the middle of the sequence to reduce possible tension at the junction of double-stranded regions with the insert, d(GGGT)_4_ or d(GT)_8_, and to facilitate G4 formation.

**Figure 5:**
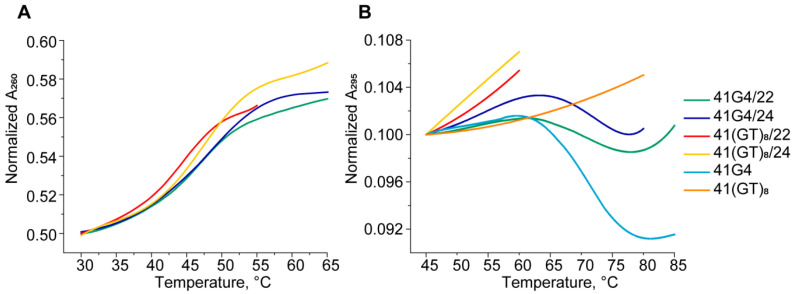
Thermal melting curves of single- and double-stranded DNA models in buffer A at ~3 µM oligonucleotide strand concentration. Melting curves for UV absorbance (**A**) at 260 nm and (**B**) at 295 nm.

At first, UV spectroscopy was performed to assess the secondary structures formed by our single- and double-stranded DNA models. The temperature dependence of UV absorbance at 295 nm is a marker of a G4 structure. Unlike a DNA duplex, where melting is accompanied by a hyperchromic effect (usually at 260 nm), melting of a G4 at 295 nm causes a decrease in the optical density, and this is a cooperative process. Furthermore, denaturation of a DNA duplex that may coexist with the G4 structure does not contribute to the G4 melting profile at this wavelength [38]. Structural domains in 41G4/22 and in several control systems that cannot form a duplex region (41G4), a G4 region (41(GT)_8_/22), or both (41(GT)_8_), were monitored independently at 295 and 260 nm (Figure 5). The melting curves of 41G4 and 41G4/22 recorded at 295 nm showed one-step cooperative transitions corresponding to G4 unfolding with observed hypochromic effects that varied from 10% for 41G4 to 3% for 41G4/22 (Figure 5B). This phenomenon is explained by the presence of additional 22 nucleotide residues in 41G4/22, which absorb the UV light but do not contribute to the G4 unfolding. Melting temperatures (*T*_m_) values of the G4 structure formed in single-stranded and double-stranded DNA species are almost equal (70–72 °C; Table 1). As expected, the melting profiles of 41(GT)_8_/22 and 41(GT)_8_, which are unable to fold into a G4 structure, manifested only a slight increase in UV absorbance at 295 nm (Figure 5B). The presence of duplex domains in the folded states of 41G4/22 and 41(GT)_8_/22 was confirmed by UV melting at 260 nm (Figure 5A). Their melting profiles show a hyperchromic effect, as opposed to the G4 unfolding profile (at 295 nm). Despite an obvious disturbance in the stacking interactions along the duplex scaffold in the insertion region, single-step conformational transitions are observed for both 41G4/22 and 41(GT)_8_/22. Given that the duplex “wings” are stabilized by the same number of base pairs with similar G/C contents, it can be assumed that the observed melting curves are a superposition of the melting of two duplex regions flanking the insert. Nevertheless, the presence of the looped unstructured d(GT)_8_ insert between two duplex domains destabilizes the duplex structure more effectively than the compact G4 does (compare the *T*_m_ values: 44 and 47 °C, respectively).

Thus, the 41G4/22 unfolding process can be described as a combination of two conformational transitions spaced apart along the temperature axis. The first transition (measured at 260 nm) with *T*_m_ of 47 °C is related to denaturing of the intermolecular duplex structure, and the second transition with *T*_m_ of 70 °C (monitored at 295 nm) belongs to intramolecular G4 melting (Table 1). The addition of TT residues in the middle of a partially complementary shorter strand opposite the G4-motif or d(GT)_8_ insert (41/24 models) causes a slight increase in duplex *T*_m_ values compared to those of the 41/22 model (Table 1).

**Figure 6:**
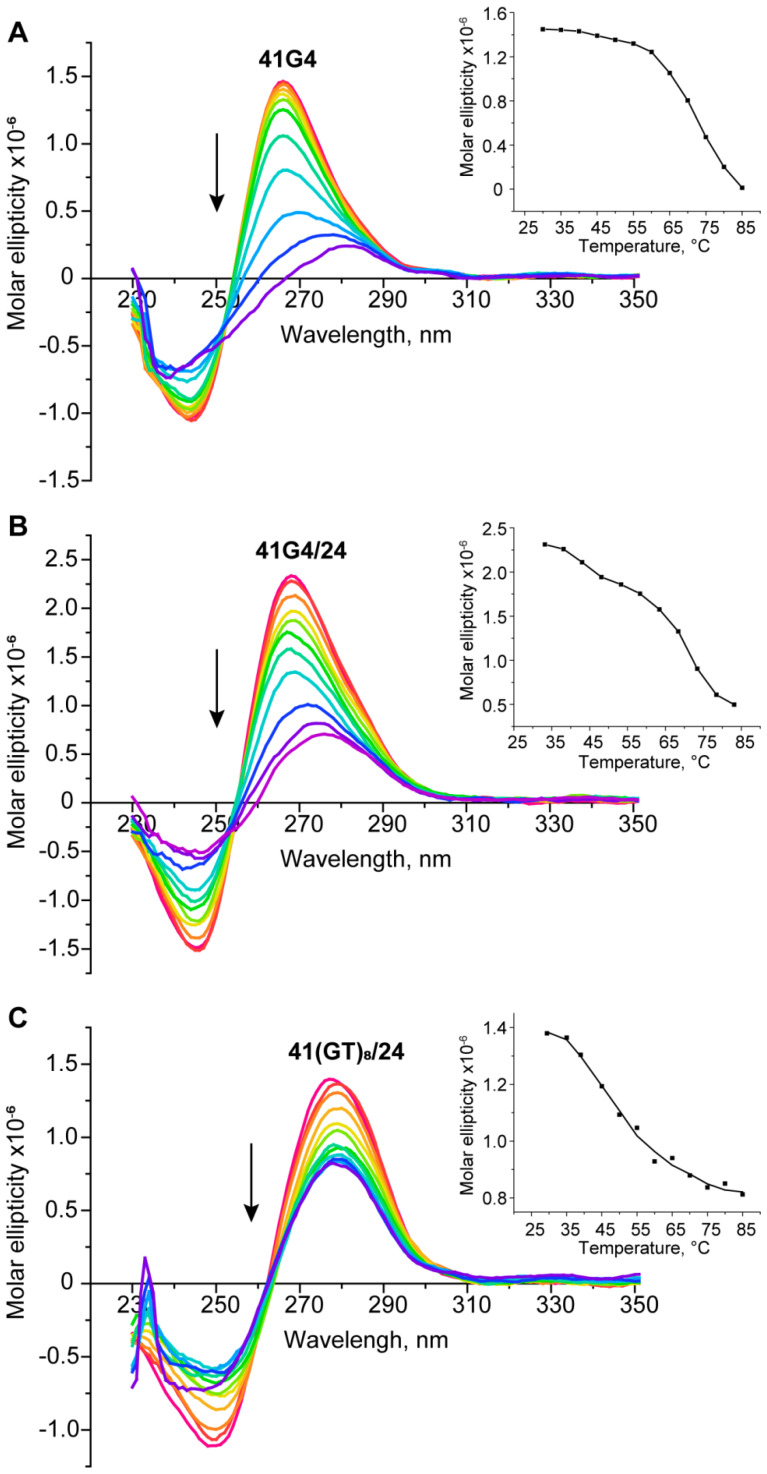
CD spectra of single- and double-stranded DNA models as recorded in buffer A at different temperatures (~2 µM oligonucleotide strand concentration). (**A**) 41G4; (**B**) 41G4/24; and (**C**) 41-(GT)_8_/24. The arrow indicates the temperature increase from 30 to 85 °C in 5 °C increments; multicolor lines show the CD spectra at different temperatures rising along the direction of the arrows. (Insets) CD-monitored melting profiles at the wavelength of an ellipticity maximum.

CD spectra of the single-stranded 41G4 oligonucleotide and of 41G4/24, in which the d(GGGT)_4_ sequence is integrated into a duplex context, revealed a typical parallel G4 fold with a positive peak at 265 nm and a negative one at 245 nm (Figure 6A,B). Of note, the addition of a partially complementary strand to 41G4 caused only small changes in the CD spectrum of 41G4/24 because the characteristic CD band of the DNA duplex domain, located at 270–280 nm, is masked by an intense positive peak corresponding to the parallel-stranded G4. On the other hand, a greater amplitude and peak maximum shift to 268 nm clearly indicates the presence of a duplex domain in 41G4/24. As expected, the CD spectra of 41(GT)_8_ and 41(GT)_8_/22 do not have typical G4 signatures (Figure 6C).

A positive peak is present at ~280 nm, where the signals of the unstructured oligonucleotides and B-DNA are located. CD spectral data recorded at different temperatures made it possible to obtain melting curves for all the studied DNA models (Figure 6, insets). As readers can see, the CD melting curve for 41G4 determined at 265 nm is in good agreement with the temperature dependence of UV absorbance at 295 nm with *T*_m_ of 70 °C, while the CD melting profile for 41(GT)_8_/24, which does not fold into a G4 structure, reflects the denaturation of the duplex domain with *T*_m_ of ~49 °C. Moreover, the CD melting curve of 41G4/24, examined at 268 nm, showed a two-step transition with *T*_m_ values corresponding to duplex domain denaturation (~43 °C) and G4 unfolding (70 °C). These values are consistent with the UV spectroscopy results, thereby confirming the independent unfolding of both structural domains.

### 2.5. Dual-Label Fluorescent DNA Probes Are Informative for Detecting Duplex Formation in 41/22 Models rather than G4 Folding

**Figure 7:**
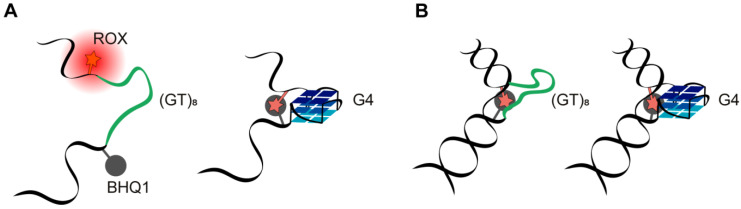
The mutual arrangement of the ROX fluorophore (red asterisk) and dark quencher BHQ1 (gray circle) attached in the immediate proximity to the ends of G4 or disordered d(GT)_8_ inserts within single- and double-stranded DNAs. (**A**) Oligonucleotides 41G4-ROX-BHQ1 and 41(GT)_8_-ROX-BHQ1. (**B**) Duplexes 41G4-ROX-BHQ1/22 and 41(GT)_8_-ROX-BHQ1/22.

In addition to conventional methods, the secondary structure of single- and double-stranded DNA models was probed by means of double-labeled 41-nt DNAs with rhodamine X fluorophore (ROX) as a fluorescent reporter and black hole quencher (BHQ1) as a quencher attached at the C5 position of internal thymine residues at 5′ and 3′ ends of the G4 motif (or control sequences), respectively (Table S1). A schematic representation of mutual arrangements of the two chromophores depending on the secondary structure elements is given in Figure 7; DNA duplex formation and/or G4 folding is believed to bring ROX and BHQ1 closer to each other, thereby causing fluorescence quenching. This method has not previously been applied for these purposes. The results obtained (Figures S1 and S2, Table S2, and corresponding annotations in the Supplementary Materials) constitute the first experimental evidence that chromophores ROX and BHQ1 can help to detect G4 formation in single-stranded DNA, although the quenching effects are rather modest. Nonetheless, this approach did not prove to be promising for G4 detection in a double-stranded context when, as in our case, the reporter and quencher moieties are in the same strand. On the other hand, it was found that ROX–BHQ1 dual-label DNA models could be applicable to the monitoring of duplex formation, regardless of the insertion sequence.

### 2.6. An Intramolecular G4 Tightly Binds MutS Regardless of a Nucleotide Cofactor and Stimulates MutS ATPase Activity

The newly designed DNA models, including those containing a parallel-stranded fold-back intramolecular G4, were next used to compare MutS-binding properties toward a mismatched base pair and G4 structure and to analyze the rate of ATP hydrolysis.

MMR is a complex and dynamic process that is usually initiated by specific binding of MutS to a DNA mismatch. MutS protein homologs are known to possess two key activities: (i) the ability to recognize and bind mismatched base pairs and distinguish a variety of non-Watson–Crick structures in DNA, and (ii) an ATPase activity that modulates MutS interactions with DNA and other proteins during initiation of MMR [39]. In bacteria, MutS operates as a homodimeric protein [40]. Briefly, mismatch recognition by ADP-bound MutS drives a rapid exchange of ADP for ATP, thus resulting in coordinated conformational changes, the formation of a sliding clamp that randomly diffuses along DNA, and the recruitment of the MutL protein (Figure S3) [41,42]. Because MutS plays a central part in the detection of and signaling responses to mismatches and to some non-B form structures within DNA, we began by comparing the binding of ecMutS (methyl-directed mechanism) and rsMutS (methyl-independent mechanism) to 76/95G4-A/T lacking a G/T mismatch but containing an intramolecular G4. The 76/76-G/T, 76/95(GT)_8_-A/T, and 76/76-A/T DNAs served as the controls (Figure 1). To gain further insights into how nucleotide cofactors affect the binding mode and stability of the DNA–MutS complex, we conducted a series of measurements in the presence of ADP, ATP, or adenosine-5′-O-(3-thiotriphosphate), which is a nonhydrolyzable ATP analog (ATPγS). Complex formation was monitored by an electrophoretic mobility shift assay (EMSA) using double-stranded DNA ligands labeled with the TAMRA fluorophore at the 3′ end of the “bottom” strand. The curves of MutS binding to DNA ligands are presented in Figure S4, and the corresponding *K*_D_^app^ values are in Table 2.

ADP bound at the ATPase site of MutS is known to contribute to the formation of a stable initial recognition complex between MutS and mismatch-containing DNA, where the DNA duplex is sharply bent at the mismatch position [43]. According to the data presented in Table 2, there are no differences in the affinity of MutS homologs, especially ecMutS, for various DNA ligands, including the G4-containing one, in the presence of ADP. In all cases, strong DNA–MutS complexes are formed. Moreover, the estimated *K*_D_^app^ values are not noticeably different between ecMutS and rsMutS.

Unlike ADP, cofactor ATPγS, as well as ATP, promote the formation of a stable MutS sliding clamp after mismatch recognition, which ultimately leads to DNA unbending and disassembly of the specific MutS complex with DNA (Figure S3) [42]. The analysis of MutS binding to ligands 76/76-G/T, 76/95(GT)_8_-A/T, and 76/76-A/T revealed an increase in *K*_D_^app^ when ATPγS is added instead of ADP (Table 2). The most dramatic decrease in MutS affinity for the above DNA ligands was demonstrated for rsMutS; *K*_D_^app^ values of the complexes between rsMutS and 76/76-G/T, 76/95(GT)_8_-A/T, or 76/76-A/T are almost threefold higher than those in the corresponding complexes with ecMutS. Remarkably, the ability of MutS from the two bacteria to bind G4-containing 76/95G4-A/T in the presence of ATPγS was almost the same as that of ADP. The retention of ecMutS affinity for G4 DNA structure upon ATPγS addition has been observed previously when an intermolecular G4 from a murine immunoglobulin switch region was used as a target [19].

Because MutS-induced conversion of ATP to ADP is Mg^2+^-dependent, the interaction between ecMutS or rsMutS and DNA was also tested at 1 mM ATP and 5 mM MgCl_2_, i.e., under conditions mimicking a cellular medium [44]. Although the MutS homologs from both bacteria showed slightly reduced affinity for all control DNA ligands as compared to those in the presence of ADP, they retained high affinity to G4-containing 76/95G4-A/T, comparable to that observed in the presence of ADP or ATPγS (Table 2). Our findings are consistent with previously published data showing that the binding of human MutSα to an isolated intermolecular G4 structure is ATP-independent [19]. Thus, the type of nucleotide cofactor does not affect the efficiency of the interaction of G4 with the MutS proteins from various sources.

Interactions of MutS with DNA during the search for mismatches, mismatch recognition, and initiation of repair are modulated by its ATPase activity. In addition, MutS is believed to function as a molecular switch involving communication between mismatch recognition and nucleotide-binding sites [42]. The binding of MutS to DNA induces a decrease in the protein’s affinity for ADP, thus facilitating the replacement of ADP with ATP at the ATPase site and, accordingly, increases the rate of ATP hydrolysis in the presence of a DNA ligand [41]. Given that the binding of a G4 to eukaryotic MutSα [19], as well as to ecMutS and rsMutS studied in this work, is reported to be independent of the nucleotide cofactor type, the ATPase activity of ecMutS mediated by each DNA ligand under study was evaluated next. To measure the stationary kinetics of ATP hydrolysis, we applied a colorimetric assay based on malachite green complex formation with phosphomolybdic acid for inorganic phosphate detection. The kinetic parameters (Michaelis constant, *K*_M_, and a catalytic rate constant, *k*_cat_) of ATP hydrolysis by the ecMutS protein were determined both in the presence and absence of DNA ligands (Table 3, Figure S5). The Michaelis constants, characterizing the ecMutS affinity for ATP, were not affected by the DNA tested, although the maximal initial rate of ATP hydrolysis by the ecMutS increased 2–3-fold in the presence of a DNA ligand, irrespective of its type, as compared to the DNA-free sample, with the highest *k*_cat_ for the mismatch-containing 76/76-G/T. The increased ATPase activity of MutS in the presence of DNA has also been observed in other studies [41]. Nevertheless, this is the first time that ecMutS binding to a G4-containing DNA duplex is shown to promote ATPase activity, as does the binding to other DNA ligands. Consequently, the peculiar mode of MutS interaction with the G4 structure (this mode manifests itself in ATP-independent DNA binding) does not affect the ability of the protein to hydrolyze ATP.

### 2.7. Highly Effective Binding of G4 DNA to ecMutL Is Discovered

Following the recognition of damaged DNA, other proteins via error-specific signals are recruited to initiate and complete repair. MutL, which serves as a molecular matchmaker (and endonuclease in most bacteria and eukaryotes), is recruited in a MutS- and ATP-dependent manner, forming ternary MutS–MutL complexes on mismatched DNA [39]. The MutL protein is less studied than MutS, owing to its dynamic structure with a variety of conformations. For a long time, the main function of MutL has been believed to be exclusively the coordination of protein–protein interactions in the subsequent steps of the repair after mismatch/lesion recognition [45]. The endonuclease function of MutLα in eukaryotes and in some bacteria was demonstrated later (in that order), implying a more significant role for MutL in MMR initiation in these organisms [46,47].

**Figure 8:**
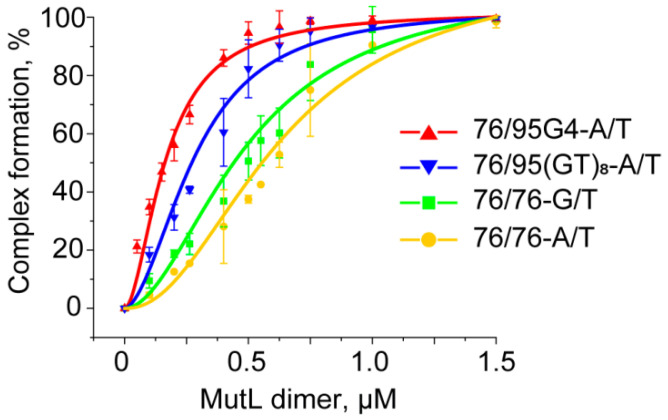
Binding of ecMutL to DNA ligands. The yield of nucleic acid–protein complexes, as calculated from the data of the EMSA, is plotted against ecMutL concentration (0–3.0 μM) at 100 nM DNA (*p* < 0.05).

MutL has weak DNA-binding activity, which is largely dependent on DNA length [48]. The size of the DNA site bound by MutLs from different species varies from 30 bp in the case of *Aquifex aeolicus* MutL [49] to somewhere between 250 and 500 bp for yeast Mlh1–Pms1 [50]. Although complexes of ecMutL with 41-bp DNA have been detected [51], the involvement of MutL from *R. sphaeroides* (rsMutL) in DNA binding remains unknown. It has been previously shown that MutL binding to DNA can be clearly observed in low-salt conditions, and this process is ATP-independent [48,50,52]. Given these data, we investigated the binding of ecMutL and rsMutL to a TAMRA-labeled 209-bp DNA duplex in the EMSA. We hoped that the DNA length of 209 bp will be long enough to assess previously unknown DNA-binding properties of rsMutL. Although 2.0–2.5 μM ecMutL formed a complex with 100 nM 209-bp DNA with 100% efficiency, no interaction between rsMutL and the same DNA sample was observed even with a 20-fold excess of the protein (Figure S6). Nevertheless, rsMutL was found to nick the pUC-MMR plasmid in the presence of certain divalent metal ions, thus proving its activity (Figure S7). The lack of rsMutL affinity for 209-bp DNA suggests that the binding of this protein to every 76/95 DNA model is highly unlikely. Therefore, we evaluated relative binding affinity of only MutL from *E. coli* for TAMRA-labeled 76/95G4-A/T, 76/76-G/T, 76/95(GT)_8_-A/T, and 76/76-A/T (Figure 8 and Figure S8). Of particular interest was the assay of ecMutL binding to a G4-containing DNA duplex because the role of G4 in MutL functioning is not clear so far. According to our data, ecMutL efficiently interacts with 76/95G4-A/T (Figure S8A). The binding extent of 0.1 μM ecMutL toward this ligand was revealed to be 1.9-fold higher than that toward a DNA duplex with an unstructured d(GT)_8_ loop, and at least 3.7-fold higher than that toward the perfect duplex 76/76-A/T and mismatched duplex 76/76-G/T (Figure 8). Nevertheless, *K*_D_^app^ of the ecMutL–76/95G4-A/T complex (~200 nM) is almost an order of magnitude higher than *K*_D_^app^ of the ecMutS complex with the same G4-containing DNA ligand (Table 2), indicating that in the presence of these two principal components of the MMR, MutS preferentially interacts with a G4 in a duplex context, at least in vitro.

### 2.8. A G4 Unlike a G/T Mismatch Does Not Activate the MMR System

**Figure 9:**
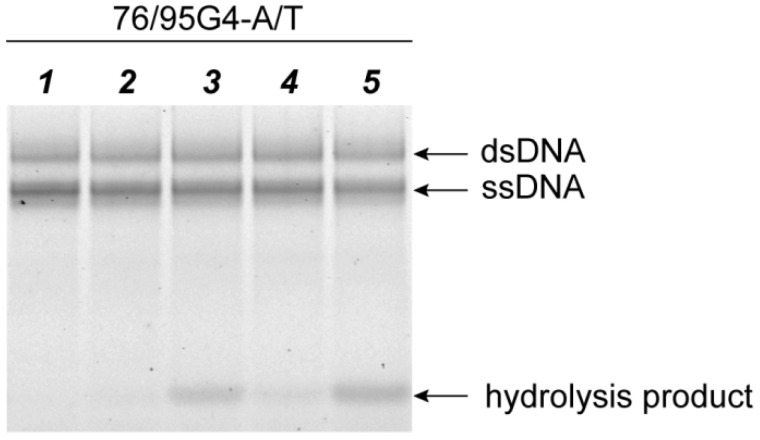
Hydrolysis of G4-containing duplex substrate 76/95G4-A/T under the influence of *E. coli* MMR proteins. Lane *1:* untreated DNA, *2:* DNA incubated with 250 nM MutH, *3:* DNA incubated with 250 nM MutH and 250 nM MutL (per monomer), *4:* DNA incubated with 250 nM MutH and 250 nM MutS (per monomer), *5:* DNA incubated with 250 nM MutH, 250 nM MutL, and 250 nM MutS (per monomer). The reaction mixtures were incubated in the presence of 1 mM ATP for 1 h and then electrophoresed in a 10% polyacrylamide gel containing 7 M urea.

The MutH protein is known to nick the unmethylated strand at a 5′-Gm^6^ATC-3′/3′-GATC-5′ monomethylated site launching a further repair process; the presence of both MutS and MutL significantly enhances the efficiency of DNA hydrolysis [53]. We studied the hydrolysis of each DNA ligand—76/95G4-A/T, 76/76-G/T, 76/95(GT)_8_-A/T, and 76/76-A/T—under the action of ecMutH. Of note, DNA ligands 76/95G4-A/T and 76/95(GT)_8_-A/T contain a MutH monomethylated recognition site, whereas G4 or d(GT)_8_ inserts are located in the unmethylated longer strand (Table S3). DNA duplexes 76/76-G/T and 76/76-A/T served as the controls. A MutH-mediated nick in the “bottom” DNA strand resulted in the formation of a 3′-labeled 18-nt product, which was separated from the nonhydrolyzed DNA ligand by gel electrophoresis under denaturing conditions, allowing for evaluation of the cleavage efficiency (Figure 9).

**Figure 10:**
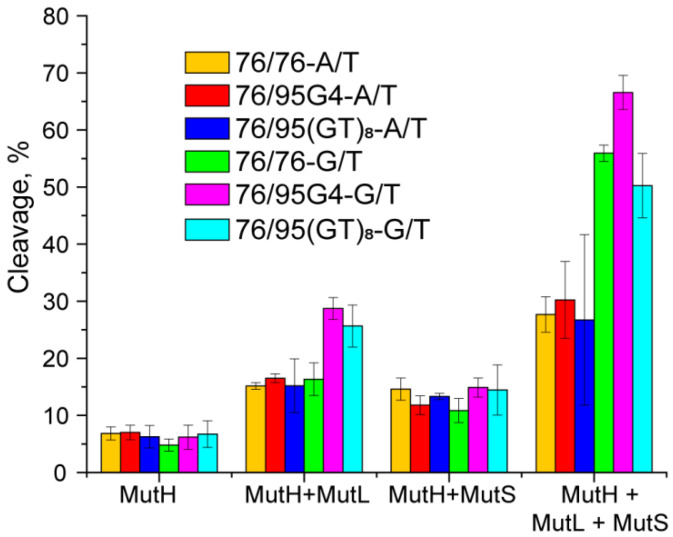
Efficiency of DNA hydrolysis induced by *E. coli* MMR proteins (*p* < 0.05). Data were obtained for endonuclease MutH alone (at a concentration of 250 nM) and for the combinations of MutH with 250 nM MutS or 250 nM MutL as well as with both 250 nM ecMutS and 250 nM ecMutL (all protein concentrations are calculated per monomer). The reaction mixtures were incubated at 37 °C for 1 h.

The DNA hydrolysis products were analyzed for ecMutH alone as well as for ecMutH in the presence of ecMutS, ecMutL, or both proteins at equal concentrations of 250 nM per protein monomer (Figure 10). ecMutH alone was not able to distinguish between the examined DNA ligands, leaving the hydrolysis efficiency equally low (~8%). ecMutS added to the reaction weakly stimulated the endonuclease activity of ecMutH, thereby causing an approximately 1.7–2.4-fold increase in the hydrolysis efficiency for each DNA ligand. A slight (2.2–2.6-fold) enhancement in ecMutH activity was also observed in the presence of ecMutL. Lastly, the efficiency of hydrolysis of 76/95G4-A/T and 76/95(GT)_8_-A/T without a mismatched base pair under the action of a complete MutS–MutL–MutH protein set did not differ from that for the perfect 76/76-A/T DNA (~30%). At the same time, DNA cleavage caused by the combined action of ecMutS, ecMutL, and ecMutH was twofold more effective for the 76/76-G/T DNA ligand containing a G/T mismatch as a cognate DNA lesion recognized and repaired by the MMR system.

### 2.9. A G4 Does not Prevent the Mismatch-Dependent Activation of E. coli MMR

The next task was to understand whether G4 in a DNA duplex context affects the functioning of MMR. Therefore, a G/T mismatch was introduced into ligands 76/95G4-A/T and 76/95(GT)_8_-A/T at a distance of 17 bp from the G4/unstructured loop (Table S3). Moreover, in the new constructs, referred to as 76/95G4-G/T and 76/95(GT)_8_-G/T, the non-B-DNA structural elements were located between the MutH cleavage site (at a distance of 19 bp from it) and a mismatched base pair. The cleavage efficiency of 76/95G4-G/T and 76/95(GT)_8_-G/T by ecMutH endonuclease alone and in the presence of ecMutL, ecMutS, or both was evaluated and compared with those of 76-bp DNA ligands 76/76-A/T and 76/76-G/T lacking G4 or d(GT)_8_ inserts. Again, ecMutH alone was shown to cleave all the examined DNA ligands with equally low efficiency, whereas ecMutS added to the reaction doubled the ecMutH endonuclease activity (Figure 10).

Moreover, 76/95G4-G/T and 76/95(GT)_8_-G/T were hydrolyzed by ecMutH in the presence of ecMutL ~4-fold more effectively than by MutH alone, whereas for other DNA ligands, this difference was half as much. Because ecMutH is known to colocalize and operate in coordination with ecMutL [39], the observed effect can be explained by the recruitment of an increased number of ecMutL molecules to structural defects (G4 or d(GT)_8_ inserts) causing the activation of additional ecMutH molecules. Finally, the combined action of the complete set of MMR proteins—ecMutS, ecMutL, and ecMutH—yields the highest efficiency of MutH-mediated cleavage (~70%) in the case of the G4-containing 76/95G4-G/T ligand. In contrast, the presence of a d(GT)_8_ loop in mismatched DNA did not affect the initiation of the MMR process, which manifested itself as DNA ligand cleavage.

## 3. Discussion

MMR is a highly efficient mechanism of DNA metabolism. MMR is required for proper maintenance of the genome by protecting against base pair mismatches and insertion/deletion loops arising from DNA polymerase errors or during homologous recombination. Overall, the majority of bacterial and eukaryotic DNA repair systems share a great deal of similarity in the ways these groups of organisms remove various types of damage from DNA. This fact is partially explained by the general uniformity of DNA damage types in all organisms [22]. The principal components of the MMR system, MutS and MutL, are highly conserved proteins that are both needed to initiate appropriate DNA repair and DNA damage signaling responses in an orchestrated manner. Eukaryotic and *E. coli* MMR pathways have two main distinctive features: (i) nick- and methyl-mediated strand discrimination mechanisms, which are provided by eukaryotic MutL homologs and ecMutH, respectively, and (ii) functioning of heterodimeric MutS and MutL eukaryotic homologs instead of homodimeric bacterial ones. Despite these differences, the MMR systems that have persisted throughout evolution are similar to a great extent, especially in terms of MutS functions. Substantially similar properties among eukaryotic MutSα (MSH2/MSH6 dimer), MutS homologs from MutH-free bacteria, and ecMutS include high affinity for DNA mismatches, the ATPase function that promotes DNA repair by stimulating MutS conformational changes, and the ability to recognize and interact with noncanonical DNAs, in particular G4 DNA. Crystal structures of the ecMutS homodimer and human MutSα bound to a DNA mismatch reveal the same asymmetric binding mode, where only one subunit is responsible for the specific interaction with a mismatch through an intercalated phenylalanine residue, indicating that ecMutS is a functional heterodimer [54]. Therefore, *E. coli* MMR may serve as a useful tool for functional studies of the eukaryotic MMR pathway.

Over the past two decades, evidence has emerged for the occurrence of G4s in the genome, their biological significance, and dual functions [8,55]. Putative G4-forming sequences are unevenly distributed across the cell genome and are mainly clustered in “hot” genomic regions involved in many important biological processes. G4 motifs have been found not only in mammalian genomes but also in yeasts [56], protozoa [57], bacteria [58,59], and viruses [60]. Although the function of G4s in prokaryotes is not fully elucidated, these non-B form structures are considered important regulators of pathogenic processes because these structures control the expression of virulence genes [61]. The presence and locations of G4-forming sequences in all bacterial genomes available in the NCBI database were analyzed via the G4Hunter algorithm in a recent paper [62]. G4 motifs were identified in all species, but their frequency differed significantly across evolutionary groups. Among loci enriched with G4 motifs, promoter regions and noncoding-RNA genes were identified. Analysis of a large number of open reading frames in 18 genomes of evolutionarily distant bacterial species has uncovered the presence of G4-forming sequences in –200 bp upstream regulatory regions for 14.7% of the genes [21]. G4 motifs in bacterial promoters are conserved for orthologous genes from different organisms; this observation confirms the regulatory functionality of G4s. In contrast to eukaryotic genomes, there is only one example of a well-documented impact of a G4 structure on pilin antigenic variation in *Neisseria gonorrhoeae* as a consequence of G4-dependent DNA recombination [63]. In *E. coli*, the formation of G4 structures has been detected in vivo [23]. High-throughput G4 sequencing analysis of the *E. coli* genome with 50.8% G/C content has identified 131 putative G4-forming sequences, among which unusual motifs with only two G-quartets were the most prevalent (68.1%) [64]. Conversely, among 1990 computationally defined G4 motifs detected in the *R. sphaeroides* genome with 68.8% G/C content, the (G_3+_N_1–7_)_4_ conventional motif with at least three G-quartets and loops of length up to seven nucleotides were dominant. The data from recent papers point to the influence of a G4 on transcription in *E. coli* [65], *Deinococcus radiodurans* [66], and *Mycobacterium tuberculosis* [67].

Along with a wide range of other G4-recognizing molecular actors, many proteins involved in DNA repair systems interact with G4 DNAs. In particular, the G4 structures that resist normal metabolic processes under physiological conditions can be unwound by a variety of special helicases (Pifl, FANCJ, BLM, WRN, and bacterial RecQ) or may even be completely removed by nucleases (e.g., DNA2). If these proteins do not function properly or are absent, several repair systems, including θ-polymerase-mediated end joining [68], homologous recombination [69], and BER, can participate in the repair of emerging non-B form DNAs. G4s have been reported to interact with XPD and XPB, which are parts of the TFIIH complex participating in transcription and NER [70], with XPD acting as a G4 helicase and XPB acting as a G4-binding protein. Recent experiments indicate an important role of APE1, a key enzyme in human BER, in the maintenance of telomere structure. In vitro experiments suggest that APE1 can bind various G4 structures and remove the apurinic/apyrimidinic sites present there [12].

Analysis of the mode of MutS binding to G4s has aroused great interest because some studies [19,20] have revealed variations in the ways that MutS from *E. coli* and its human homolog interact with quadruplex structures, on the one hand, and DNA mismatches, on the other. Ehrat and coauthors have proposed that MutS is unable to activate the ATP-dependent canonical MMR pathway through G4 binding and that the function of MutS in G4 DNA metabolism is not associated with methyl-directed MMR. Direct evidence supporting this hypothesis has not yet been provided. In the current study, we analyzed the involvement of G4 recognition by MutS proteins from two bacteria, γ-proteobacterium *E. coli* (methyl-directed MMR) and α-proteobacterium *R. sphaeroides* (methyl-independent MMR inherent in eukaryotic organisms), in a cascade of subsequent events. For the first time, we evaluated the contribution of G4s to the functioning of other proteins involved in the initial stage of *E. coli* MMR. Accordingly, the main objective of our study was to answer the question of whether G4 binding to MutS blocks the action of endonuclease ecMutH or triggers a series of regulatory responses that affect the next steps of MMR.

For this purpose, a simple model in the form of an isolated G4, which has been used in previous studies, was unsuitable. A biologically relevant intramolecular G4 should be embedded into a DNA duplex containing the entire set of elements necessary for the initiation of MMR (a G/T mismatch and MutH recognition site). In accordance with these requirements, we created a novel DNA model containing the G4 structure formed by the d(GGGT)_4_ sequence in a “frozen” state in a DNA duplex context (Figure 1). The G4 folding in this case did not require negative supercoiling, facilitating non-B-DNA formation in the genome. According to the literature data [28,71,72], the d(GGGT)_4_ sequence has a potential to fold into a topologically homogeneous three-layered parallel-stranded G4 with propeller one-nucleotide loops. Extremely high thermodynamic stability that preserves the G4 structure even in the presence of the complementary strand is a hallmark of this quadruplex [73]. The d(GGGT)_4_ sequence has been shown to be widespread in genomic DNA and is recognized by many proteins in eukaryotic cells and viruses, such as the STAT3 protein, topoisomerase 1 [28,74], interleukin-6 receptor, and HIV-1 integrase [75]. Notably, G4-motifs, which are located in promoter regions, mainly fold into parallel G4s (for a review, see reference [9]). Apparently, G4-recognizing proteins involved in the regulation of cell proliferation, DNA replication, and transcription preferentially recognize this type of quadruplex topology. A very similar sequence that folds into a parallel-stranded G4 constitutes a recombination initiation site in the bacterium *N. gonorrhoeae* [63].

The secondary structures of the newly designed single- and double-stranded DNA models containing a G4-motif or the unstructured d(GT)_8_ insert together with the control DNAs lacking the looped area (Figure 1) were characterized by various biophysical and biochemical techniques.

The G4 and duplex structures within the long DNA constructs were evaluated through examination of imino proton NMR spectra. The advantage of the NMR approach is its ability to identify signals of the exchangeable imino protons characteristic of Watson–Crick and Hoogsteen base pairing in a single experiment. According to our **^1^**H NMR data, the d(GGGT)_4_ insert assumes the G4 conformation in both single- and double-stranded DNA contexts, whereas DNA duplex- and G4 domains coexist in the secondary structure of 76/95G4-G/T (Figure 2). G-quartet guanines’ poorly resolved **^1^**H imino resonances, which complicate detailed structural analysis, have been previously described for d(GGGT)_4_ and related oligonucleotides [76] and are attributed to the quasi-symmetry of the structure arising from the repetitive nature of the sequences.

As an independent method for direct monitoring of G4 and duplex domains in the DNA models under study, we utilized chemical probing assays with DMS and KMnO_4_. G_3_ tracts in 76/95G4-A/T manifested reduced reactivity toward DMS, as compared with other guanines in the rest of the sequence, thus revealing the G4 structure formed by the d(GGGT)_4_ insert. In contrast, GT repeats in 76/95(GT)_8_-A/T showed no evidence of G4 formation in experiments with DMS- and KMnO_4_-induced modifications (Figure 3) [76]. The attenuation in the KMnO_4_ reaction corresponded to duplex regions in samples 76/95G4-A/T and 76/95(GT)_8_-A/T. In general, the results of both ^1^H NMR spectroscopy and chemical probing prove the formation of an intramolecular G4 structure stabilized in a double-stranded context.

These findings are consistent with the CD and UV spectroscopy data that were obtained with shorter 41/22(24) DNA analogs (Figure 4) to improve the signal-to-noise ratio. As evident from the CD spectra, the d(GGGT)_4_ insert in both single- and double-stranded contexts has the characteristic features of a parallel G4 folding pattern wherein all the guanosines in the G-quartets are in an anti-conformation (Figure 6). The parallel topology is known to be predominant for G4s with three one-nucleotide loops [28,76,77]. Using UV spectroscopy, which enables independent monitoring of G4 and DNA duplex unfolding at 295 and 260 nm, respectively, we estimated *T*_m_ values of both structural domains: intramolecular G4, whose thermal stability was practically the same (*T*_m_ is ~71 °C) in single- or double-stranded contexts, and an intermolecular DNA duplex. *T*_m_ values range from 44 to 48 °C depending on the insert sequence (d(GGGT)_4_ or unstructured d(GT)_8_) and on the length of the oligonucleotide (22 or 24 nt) complementary to the regions upstream and downstream of the insert (Table 1, Figure 5). Not surprisingly, *T*_m_ values of G4 in 41G4/22 and 41G4/24 were almost the same as *T*_m_ in single-stranded 41G4 because the G4-containing oligonucleotide released after duplex denaturation (at approximately 46 °C) is identical to 41G4.

The designed DNA models allowed us to prove that ecMutS specifically binds to the biologically relevant intramolecular G4 structure embedded into a DNA duplex, with stronger affinity than the affinity for a G/T-mismatched base pair in the presence of a nonhydrolyzable ATP analog (Figure S3). These results are consistent with previously described ATPγS-independent binding of ecMutS to isolated intermolecular G4 DNA [20], thereby confirming the validity of the latter. Ehrat and coauthors have hypothesized that MutS is unable to activate the ATP-dependent canonical MMR pathway in response to G4 recognition. Using more advanced G4 models, we found experimental evidence that the modes of ecMutS binding to mismatch- and G4-containing DNAs are different. We found that ATP- and ATPγS-independent binding of bacterial MutS homologs to a G4 inserted into the DNA duplex is not unique for ecMutS because rsMutS (involved in methyl-independent MMR) also specifically interacts with the extrahelical G4 (Table 2). Moreover, the binding of G4 DNA to ecMutS promoted ATPase activity, just as other DNA ligands did. Thus, the mode of MutS binding to intermolecular and intramolecular G4s seems to be common among different organisms, regardless of the strand discrimination mechanism.

The following stages of MMR were examined only for the *E. coli* MMR system because rsMutL, possessing the endonuclease function, did not interact with the DNA samples used in our experiments, probably owing to the insufficient length of DNA ligands, which cannot form a stable DNA–protein complex. Despite efficient binding of ecMutS and ecMutL to G4-containing DNA, we did not find that these MMR proteins activate MutH-induced hydrolysis of 76/95G4-A/T, which does not contain a G/T mismatch (Figure 10). Nevertheless, the globular structure of G4, which necessarily causes local discontinuities in a linear DNA double helix, could interfere with the MMR process; for example, a G4, when recognized as DNA damage, may trigger the MMR process or could be resolved by MMR machinery. To clarify this issue, we reconstructed in vitro the conditions for the hydrolysis of a monomethylated 76-bp DNA duplex, in which an extrahelical G4 was present along with a mismatched G/T base pair, and compared the cleavage magnitudes for 76/95G4-G/T and corresponding control DNA duplexes with and without a G/T mismatch. It was found that the intramolecular parallel G4 stabilized in a double-stranded DNA is not perceived by the *E. coli* MMR as a signal for repair. At the same time, this non-B form structure does not prevent mismatch-dependent activation of MMR when the G4 structure and G/T mismatch are located 17 bp apart. Further experiments are necessary to comprehend the impact of the G4 located in immediate vicinity of a G/T mismatch. Either way, it is clear that the participation of *E. coli* proteins MutS and MutL in cellular responses to G4 DNA is not associated with methyl-directed MMR.

## 4. Materials and Methods

### 4.1. DNA Oligonucleotides

All oligodeoxyribonucleotides (synthesized via standard phosphoramidite chemistry and purified by high-pressure liquid chromatography on Syntol, Russia) were used without further purification. Oligonucleotide strand concentrations were determined spectrophotometrically by means of extinction coefficients derived from the nearest-neighbor data [78].

### 4.2. DNA Duplexes

DNA duplexes were prepared by annealing complementary DNA strands (by heating at 95 °C for 3 min and slowly cooling to 4 °C) in 20 mM Tris–HCl buffer (pH 8.0) with 1 mM EDTA and 100 mM KCl, unless specified otherwise; the unlabeled strand was used in a 5% excess relative to a tetramethylrhodamine (TAMRA)-labeled strand. Linear 209-bp DNA was prepared by PCR amplification using 5′-TAMRA-labeled primers 5′-TAAATTGCTAACGCAGTCAGGCACC-3′ and 5′-AATAACTAGCATAACCCCTTGGGGC-3′ and plasmid pUC-MMR as a template. The PCR product was purified with the GeneJET PCR Purification Kit (K0702, Thermo Fisher Scientific, Waltham, MA, USA).

### 4.3. Purification of Recombinant Proteins

Proteins MutS, MutL, and MutH from *E. coli* as well as MutL from *R. sphaeroides* were purified as described previously [24,79]. RsMutS was obtained for the first time in this work. The *rsMutS* gene was amplified by PCR from *R. sphaeroides* (strain 2.4.1) genomic DNA as a template. The primers, 5′-AAACTGATCACGCAACATGATGAACAAGCATG-3′ and 5′-ATTAGCATATGAGCGACGACACCGTCA-3′, contained recognition sites (underlined) for restriction endonucleases BclI and NdeI, respectively. The resulting amplicon was treated with BclI/NdeI and then cloned into the pET15b vector. The recombinant proteins with a His_6_ tag at the N terminus were expressed in *E. coli* strain BL21(DE3) and purified by Ni-NTA affinity chromatography followed by size exclusion chromatography on a Superdex 200 TM 10/300 (GE17-5175-01, GE Healthcare, Chicago, IL, USA) or an Enrich SEC 650 column (7801650, BioRad, Hercules, CA, USA) on an Äkta Purifier (GE Healthcare, Chicago, IL, USA). The resultant proteins were aliquoted and stored in 10 mM HEPES buffer (pH 7.9) with 200 or 300 mM KCl, 1 mM EDTA, 10% (*v*/*v*) glycerol, and optionally, 1 mM 2-mercaptoethanol at −80 °C. Total protein concentrations were determined via spectrophotometry at 280 nm. Extinction coefficients were calculated with the help of the service [80].

### 4.4. ^1^H NMR Spectroscopy

^1^H NMR spectra of ~0.2 mM DNA samples (Figure 1, Table S3) annealed in 10 mM potassium phosphate buffer (pH 8.0) containing 50 mM KCl were recorded on a Bruker AVANCE 600 MHz spectrometer (Ettlingen, Germany) at 25 °C. D_2_O was added to 10%. The Watergate pulse sequence was utilized for the suppression of H_2_O resonance. The ^1^H NMR spectra were processed in the Mnova software (Mestrelab Research, Santiago de Compostela, Spain).

### 4.5. A Footprinting Assay

Chemical modification of 3′-TAMRA–labeled single- or double-stranded DNA models (Figure 1, Table S3) with DMS or KMnO_4_ was performed according to the standard procedure. Namely, 20 pmol of single- or double-stranded DNA, annealed in the presence of 100 mM KCl, was incubated for 2 min at 25 °C with 7 nmol of DMS in 100 μL of 50 mM sodium cacodylate buffer (pH 8.0) containing 100 mM KCl and 0.5 mg/mL tRNA for guanine modification or for 5 min at 25 °C with 0.3 nmol of KMnO_4_ in 100 μL of a 100 mM NaOAc solution containing 100 mM KCl and 0.5 mg/mL tRNA for thymine modification. The reaction was stopped by the addition of 20 μL of 10% 2-mercaptoethanol in a 100 mM NaOAc solution. The modified DNAs were precipitated by means of an ethanol–NaOAc solution and cleaved with 10% piperidine at 90 °C for 30 min. After evaporation of piperidine, the cleavage products were dissolved in 70% formamide and separated by electrophoresis in a 20% polyacrylamide gel containing 7 M urea. DNA fragments were visualized with Typhoon FLA 9500 (GE Healthcare, Chicago, IL, USA).

### 4.6. UV Melting Experiments

Oligonucleotides containing the G4 motif (or a d(GT)_8_ insert) with single-stranded flanks (Figure 4, Table S4) were annealed in 20 mM Tris–HCl buffer (pH 7.3) containing 140 mM NaCl and 5 mM KCl (buffer A) to enable G4 formation. Direct annealing of an equimolar mixture of partially complementary oligonucleotide components (Figure 4, Table S4) in buffer A was used to prepare double-stranded DNA models. Absorbance versus temperature profiles of DNA samples (at ~3 µM concentration per oligonucleotide strand) were recorded in a 600-μL quartz microcuvette (Hellma Analytics, Germany) with an optical path length of 10 mm on a double-beam Hitachi U-2900 UV/visible spectrophotometer (Japan) equipped with a Hitachi thermoelectric controller. Changes in absorbance were monitored between 10 and 85 °C at 295 or 260 nm at a heating rate of 0.5 °C/min. *T*_m_ defined as the temperature of the mid-point, was estimated from a maximum/minimum value of the first derivative of the fitted curve for data smoothed with the Savitzky–Golay filter.

### 4.7. CD Measurements

The procedure of sample preparation for CD measurements was the same as that for the UV melting experiments. CD spectra of single- or double-stranded DNA models (Figure 4, Table S4) were recorded in a quartz cuvette of 10-mm optical path length between 30 and 85 °C in temperature intervals of ~4 °C at the average heating rate of 0.5 °C/min on a Chirascan CD spectrometer (Applied Photophysics Ltd., Surrey, UK) equipped with a thermoelectric controller. The DNA concentration was chosen to attain absorption of 0.5–0.6 at 260 nm, which gives an optimum signal-to-noise ratio. The measurements were performed in the 230–325 nm wavelength range at a scanning speed of 30 nm/min and a signal averaging time of 2 s with a constant flow of dry nitrogen. All the CD spectra were baseline-corrected for signal contributions caused by the buffer. CD spectra were plotted as molar ellipticity per oligonucleotide strand against wavelength. The spectra were processed with Origin 8.0 software using the Savitzky–Golay filter. The CD melting profiles revealed the temperature dependence of a CD signal at a specific wavelength.

### 4.8. A Fluorescence Experiment

Forty-one-nucleotide single-stranded DNAs at 300 nM (Table S1) carrying the rhodamine X fluorophore (ROX) and, in some cases, black hole quencher BHQ1, were annealed in a buffer (10 mM Tris–HCl (pH 8.0), 0.1 mM EDTA, and 100 mM KCl) that favors G4 formation. Annealing of 300 nM 41/22 DNA duplexes labeled with ROX and BHQ1 (Table S1) was performed in the same manner. Fluorescence emission spectra (λ_ex_ = 580 nm) of the prepared DNA models in the range of 600–700 nm were recorded on a single-beam Hitachi 650-10S fluorescence spectrophotometer.

### 4.9. DNA-Binding Activity of EcMutS and RsMutS

An electrophoretic mobility shift assay was employed to analyze the complex formation of the MutS protein with each DNA probe (Figure 1, Table S3). Each DNA (5 nM) labeled with the TAMRA fluorophore in 20 mM HEPES–KOH buffer (pH 7.9) containing 5 mM MgCl_2_, 120 mM KCl, 0.5 mg/mL BSA, and 1 mM nucleotide cofactor (ADP, ATP, or ATPγS) was incubated with 2.5–100 nM (per dimer) MutS at 37 °C for 15 min. Free DNA and DNA complexed with MutS were separated by electrophoresis in a 6% polyacrylamide gel under nondenaturing conditions at 4 °C. Relative intensity of the DNA bands on electropherograms obtained with Typhoon FLA 9500 (GE Healthcare, IL, USA) was evaluated in the TotalLab TL120 software. The percentage of DNA in a DNA–protein complex was determined as the ratio of the fluorescence intensity corresponding to the band of the MutS–DNA complex to the total fluorescence intensity of labeled DNA. The apparent dissociation constants (*K*_D_^app^) corresponding to the MutS concentration at which 50% of the DNA ligand was complexed with the protein were averaged for at least three independent experiments. Error is presented as 95% confidence intervals.

### 4.10. Colorimetric Determination of EcMutS ATPase Activity

We carried out an assay based on measuring the absorbance of the phosphomolybdate–malachite green complex, which has been described previously [81]. The reaction mixtures in 40 µL of 10 mM HEPES–KOH buffer (pH 7.5) containing 5 mM MgCl_2_, 120 mM KCl, ATP in various concentrations (0–700 µM), 500 nM DNA (Figure 1, Table S3) (if needed), and 250 nM MutS (per dimer) were incubated for 2 min at 37 °C. The reaction was stopped by the addition of EDTA to a final concentration of 45 mM. Thereafter, 30 µL of each sample was added to 80 µL of a freshly prepared reagent for the phosphate determination, which contained malachite green (0.0812%, *w*/*v*), polyvinyl alcohol (2.32%, *w*/*v*), ammonium molybdate (5.72%, *w*/*v*, in 6 M HCl), and water, mixed in a ratio of 2:1:1:2, in a 96-well plate. Furthermore, 2 µL of 0.1 mM K_2_HPO_4_ was added into each well to increase sensitivity. After 10 min incubation at room temperature, 20 µL of a 25% (*w*/*v*) sodium citrate solution was added. The absorbance was measured at 590 nm using a Victor X5 plate reader (PerkinElmer, Waltham, MA, USA). Each experiment was conducted at least three times. Error is presented as 95% confidence intervals. Concentrations of phosphate ions were calculated from a standard curve of a correlation between solution absorbance and phosphate concentration (5–40 µM). The kinetic analysis of MutS ATPase activity was carried out via nonlinear regression fitting of the experimental points to the Michaelis–Menten equation.

### 4.11. DNA-Binding Activity of EcMutL

DNA probes (100 nM; Figure 1, Table S3) were incubated for 10 min on ice with 50–1500 nM ecMutL (per dimer) in 20 mM HEPES buffer (pH 8.0) containing 100 mM KCl and 1 mM DTT and were electrophoresed in a 6% polyacrylamide gel under nondenaturing conditions at 4 °C. The efficiency of protein–DNA complex formation was evaluated in the same way as in the assay of the DNA-binding activity of MutS. A fluorescently labeled 209-bp PCR product at 100 nM concentration was incubated on ice for 10 min with 0–5 μM ecMutL or rsMutL in 10 mM HEPES buffer (pH 8.0) containing 100 mM KCl and 1 mM DTT. The DNA–protein complex formation was visualized in a 6% nondenaturing polyacrylamide gel.

### 4.12. Hydrolysis of Plasmid DNA by RsMutL

This method has been described earlier [24]. Briefly, 10 nM pUC-MMR plasmid was incubated with 250 nM rsMutL (per dimer) in 10 mM HEPES–KOH buffer (pH 8.0) containing 100 mM KCl and 5 mM MgCl_2_ or MnCl_2_ at 37 °C for 1 h. The reaction was terminated by the addition of 50 mM EDTA and 10 activity units of proteinase K with subsequent incubation of the mixture for 30 min at 37 °C. In control experiments, the pUC-MMR plasmid was incubated with R.BamHI (restriction endonuclease) or Nt.Bpu10I (nicking endonuclease) for 60 min at 37 °C in 10 μL of the buffer recommended for each enzyme. The reaction products were analyzed by electrophoresis in a 1% agarose gel containing ethidium bromide. The gels were photographed, and the images were processed in the Image Lab software (Bio-Rad, Hercules, CA, USA).

### 4.13. DNA Cleavage by E. coli MMR Proteins

Nicking endonuclease activity of MutH was assayed by incubating 25 nM 3’-TAMRA–labeled DNA substrate (Figure 1, Table S3) with 250 nM MutH in 20 mM HEPES–KOH buffer (pH 7.9) containing 5 mM MgCl_2_, 120 mM KCl, 0.5 mg/mL BSA, and 1 mM ATP in the presence of 125 nM MutS and/or 125 nM MutL (per dimer) or without MutS and MutL at 37 °C for 1 h. The reactions were stopped by proteinase K treatment for 15 min at 50 °C. The products of DNA hydrolysis were analyzed in a 10% polyacrylamide gel containing 7 M urea. The cleavage efficiency was calculated from at least three independent experiments by division of the intensity of a product band by total fluorescence intensity. Error is presented as 95% confidence intervals.

## 5. Conclusions

Herein, we describe a novel type of DNA ligands/substrates for the study of multicomponent G4-mediated biological processes. Their characteristic feature is the stabilization of a biologically relevant intramolecular G4 of interest within a long DNA duplex structure containing selected functional moieties (e.g., recognition sites, selectively modified sites, and signaling sequences). Our constructs are a somewhat simplistic model of G4 structure in a DNA duplex context, but compared to previously tested isolated G4s, they are much closer to the in vivo state. The general and straightforward design and synthesis make the new constructs available for a wide range of applications. We assessed a new type of DNA ligand, whose secondary structure was characterized here by various unrelated biophysical methods, to clarify the G4-mediated responses of both methyl-directed and methyl-independent DNA MMR systems. Our data showed strong binding affinities of proteins MutS and MutL for the parallel G4 structure embedded in a DNA duplex. Nonetheless, the G4-binding properties did not correlate with MMR activity. In particular, we found that a G4 is not perceived by *E. coli* MMR as damage to be repaired; at the same time, this noncanonical DNA structure does not prevent mismatch-dependent activation of MMR when the G4 and a G/T mismatch are together present in the DNA substrate at a distance of at least 17 bp.

Our findings add to the understanding of the relations among a G4′s capacity to bind MMR proteins, MMR activities, and functional consequences for genome stability. These results also expand the repertoire of G4-based aptamers as molecular tools for multifaced molecular biological research.

## Figures and Tables

**Table 1:** *T*_m_ values determined from differential UV melting curves for a G4 (at 295 nm) and DNA duplex (at 260 nm) domains in buffer A.

DNA Samples	*T*_m_ of DNA Duplex, °C ± 1	*T*_m_ of G4, °C ± 1
41G4	-^a^	71
41(GT)_8_	-^a^	-^b^
41G4/22	47	70
41(GT)_8_/22	44	-^b^
41G4/24	48	72
41(GT)_8_/24	48	-^b^

^a^ Not determined. ^b^ No evidence of G4 formation.

**Table 2:** Apparent dissociation constants (*K*_D_^app^, nM) for complexes of ecMutS and rsMutS with TAMRA-labeled 76-bp DNA ligands in the presence of ADP, ATP, or ATPγS.

DNA	ecMutS			rsMutS		
	ADP	ATP	ATPγS	ADP	ATP	ATPγS
76/95G4-A/T	23 ± 5	32 ± 4	31 ± 4	23 ± 3	23 ± 3	46 ± 5
76/76-G/T	35 ± 3	38 ± 4	70 ± 10	27 ± 4	60 ± 10	360 ± 60
76/95(GT)_8_-A/T	32 ± 3	44 ± 6	80 ± 10	31 ± 3	43 ± 5	190 ± 20
76/76-A/T	38 ± 4	50 ± 10	150 ± 20	51 ± 4	80 ± 30	470 ± 70

**Table 3:** Michaelis–Menten equation parameters for the ATP hydrolysis reaction by ecMutS in the presence of DNA cofactors.

DNA	*K*_M_, µM	*k*_cat_, min^−1^
76/95G4-A/T	47 ± 13	17 ± 1
76/76-G/T	39 ± 8	22 ± 1
76/95(GT)_8_-A/T	30 ± 9	12 ± 1
76/76-A/T	27 ± 8	15 ± 1
No DNA	35 ± 15	7 ± 1

## Supplementary Materials

Supplementary Materials can be found at https://www.mdpi.com/1422-0067/21/22/8773/s1.
