# Supplementary material for: Responses of DNA Mismatch Repair Proteins to a Stable G-Quadruplex Embedded into a DNA Duplex Structure
Source: Int J Mol Sci. 2020 Nov 20;21(22):8773. doi: 10.3390/ijms21228773 (PMC7699706; doi:10.3390/ijms21228773)
Supplement: Supplementary file 1 [file ijms-21-08773-s001.pdf]

Table S1. Oligonucleotides modified with ROX fluorophore and BHQ1 quencher. The dyes were attached to the C5 of thymine residues via 5-[N-((1-aminopropyl)-1H-1,2,3-triazol-5-yl)-hexyn-1-yl] linker (ROX) or 5-[N-(6-aminohexyl)-3-E-acrylamido] linker (BHQ1). The structures of modified thymidines are given under the table *.

Oligonucleotides	Sequence (5′-3′)
41G4-ROX-BHQ1	GTTACTCGAGCT(ROX-dT)GGGTGGGTGGGTGGG(BHQ1-dT)TATATGGCTCAG
41G4-ROX	GTTACTCGAGCT(ROX-dT)GGGTGGGTGGGTGGGTTATATGGCTCAG
41(GT)$_8$-ROX-BHQ1	GTTACTCGAGCT(ROX-dT)GTGTGTGTGTGTGTG(BHQ1-dT)TATATGGCTCAG
41(GT)$_8$-ROX	GTTACTCGAGCT(ROX-dT)GTGTGTGTGTGTGTGTTATATGGCTCAG
41T-ROX	GTTACTCGAGCT(ROX-dT)TTTTTTTTTTTTTTTTTATATGGCTCAG
22	CTGAGCCATATGCTCGAGTAAC

*

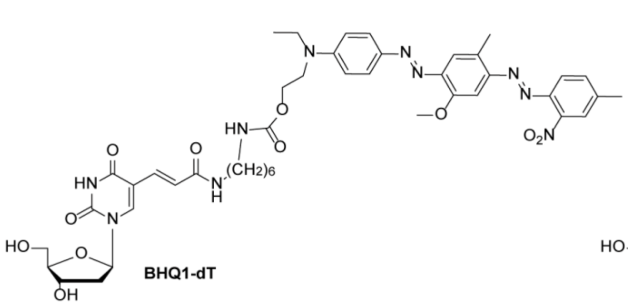

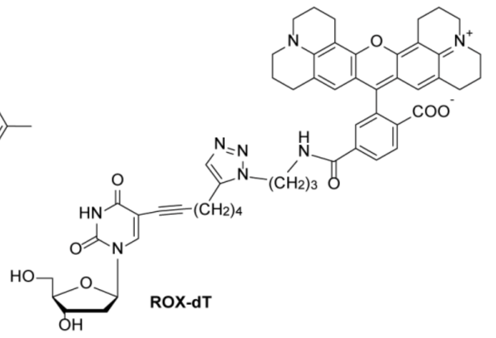

Table S2. Fluorescence intensity (in arbitrary units, a.u.) of ROX- or ROX-BHQ1-labeled 41-nt oligonucleotides with G4-motif or unordered $(GT)_8$ insert, as well as of their hybridization products with 22-nt partially complementary strand.

**Oligonucleotides**	**Fluorescence intensity at 611 nm, a.u.**
41G4-ROX-BHQ1	32±2
41G4-ROX	330±2
41$(GT)_8$-ROX-BHQ1	70±1
41$(GT)_8$-ROX	520±5
41T-ROX	510±6
**DNA duplexes**	
41G4-ROX-BHQ1/22	9±1
41G4-ROX/22	94±1
41$(GT)_8$-ROX-BHQ1/22	8±1
41$(GT)_8$-ROX/22	91±2
41T-ROX/22	93±2

Table S3. Primary structures of DNA models used to study the interactions of G4 and MMR proteins. Designations for DNA duplexes are shown on the left, and ones for single-stranded oligonucleotides are presented on the right. The sequence 5'-G**m⁶A**TC-3'/3'-CTAG-5' is the MutH recognition site.

DNA duplexes	Sequence	Oligonucleotides
76/95G4-A/T	5′-TCCTTTCGCGCTTGG**m6A**TCCTATGAGCGTTACTCGAGCA-------------------TATGGCTCAGCTGCCA**A**GCACCAGTGTCAGCGTCCTAT-3'<br>TAMRA-3′-AGGAAAGCGCGAACC--TAGGATACTCGCAATGAGCTCGTT**GGGTGGGTGGGTGGGT**TTATACCGAGTCGACGGT**T**CGTGGTCACAGTCGCAGGATA-5′	76A<br>95G4
76/95G4-G/T	5′-TCCTTTCGCGCTTGG**m6A**TCCTATGAGCGTTACTCGAGCA-------------------TATGGCTCAGCTGCCA**G**GCACCAGTGTCAGCGTCCTAT-3'<br>TAMRA-3′-AGGAAAGCGCGAACC--TAGGATACTCGCAATGAGCTCGTT**GGGTGGGTGGGTGGGT**TTATACCGAGTCGACGGT**T**CGTGGTCACAGTCGCAGGATA-5′	76G<br>95G4
76/95(GT)$_8$-A/T	5′-TCCTTTCGCGCTTGG**m6A**TCCTATGAGCGTTACTCGAGCA-------------------TATGGCTCAGCTGCCA**A**GCACCAGTGTCAGCGTCCTAT-3'<br>TAMRA-3′-AGGAAAGCGCGAACC--TAGGATACTCGCAATGAGCTCGTT**GTGTGTGTGTGTGTGT**TTATACCGAGTCGACGGT**T**CGTGGTCACAGTCGCAGGATA-5′	76A<br>95(GT)$_8$
76/95(GT)$_8$-G/T	5′-TCCTTTCGCGCTTGG**m6A**TCCTATGAGCGTTACTCGAGCA-------------------TATGGCTCAGCTGCCA**G**GCACCAGTGTCAGCGTCCTAT-3'<br>TAMRA-3′-AGGAAAGCGCGAACC--TAGGATACTCGCAATGAGCTCGTT**GTGTGTGTGTGTGTGT**TTATACCGAGTCGACGGT**T**CGTGGTCACAGTCGCAGGATA-5′	76G<br>95(GT)$_8$
76/76-A/T	5′-TCCTTTCGCGCTTGG**m6A**TCCTATGAGCGTTACTCGAGCATATGGCTCAGCTGCCA**A**GCACCAGTGTCAGCGTCCTAT-3'<br>TAMRA-3′-AGGAAAGCGCGAACC--TAGGATACTCGCAATGAGCTCGTATACCGAGTCGACGGT**T**CGTGGTCACAGTCGCAGGATA-5′	76A<br>76T
76/76-G/T	5′-TCCTTTCGCGCTTGG**m6A**TCCTATGAGCGTTACTCGAGCATATGGCTCAGCTGCCA**G**GCACCAGTGTCAGCGTCCTAT-3'<br>TAMRA-3′-AGGAAAGCGCGAACC--TAGGATACTCGCAATGAGCTCGTATACCGAGTCGACGGT**T**CGTGGTCACAGTCGCAGGATA-5′	76G<br>76T

Table S4. Primary structures of DNA models used for spectroscopic studies. Designations for DNA duplexes are shown on the left, and ones for single-stranded oligonucleotides are presented on the right.

DNA duplexes	Sequence	Oligonucleotides
41G4/22	5′-GTTACTCGAGCTT**GGGTGGGTGGGTGGG**TTATATGGCTCAG-3'<br>3′-CAATGAGCTCG-------------------TATACCGAGTC-5′	41G4<br>22
41G4/24	5′-GTTACTCGAGCTT**GGGTGGGTGGGTGGG**TTATATGGCTCAG-3'<br>3′-CAATGAGCTCG---------TT--------TATACCGAGTC-5′	41G4<br>24
41(GT)$_8$/22	5′-GTTACTCGAGCTT**GTGTGTGTGTGTGTG**TTATATGGCTCAG-3'<br>3′-CAATGAGCTCG-------------------TATACCGAGTC-5′	41(GT)$_8$<br>22
41(GT)$_8$/24	5′-GTTACTCGAGCTT**GTGTGTGTGTGTGTG**TTATATGGCTCAG-3'<br>3′-CAATGAGCTCG---------TT--------TATACCGAGTC-5′	41(GT)$_8$<br>24

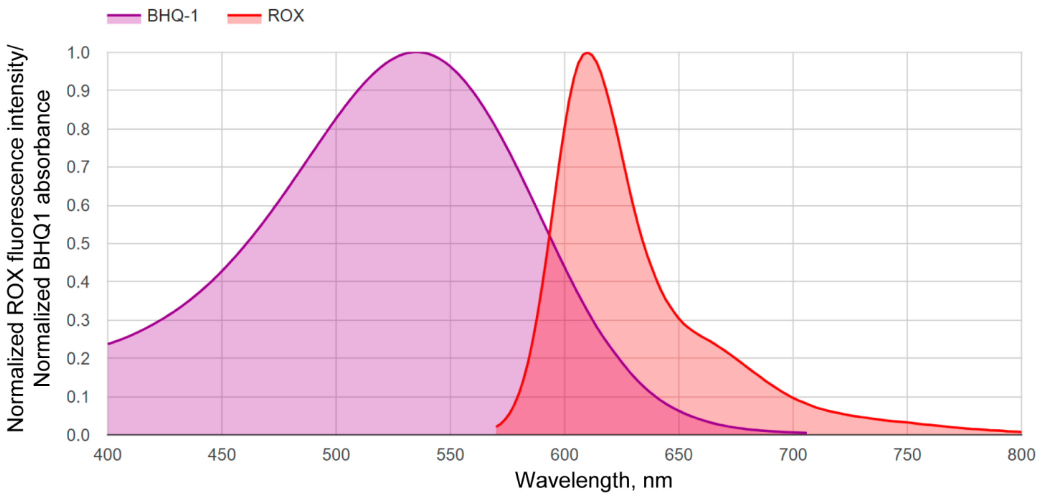

Figure S1. Overlay of fluorescence emission spectrum of ROX fluorophore and absorption spectrum of BHQ1 quencher (https://www.biosearchtech.com/qpcr-multiplex-spectral-overlay-tool).

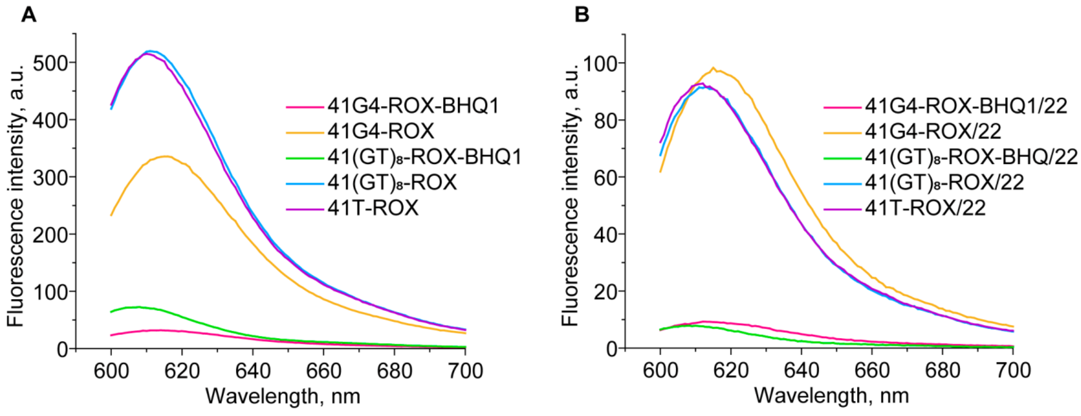

Figure S2. Fluorescence emission spectra. (**A**) ROX-labeled or ROX-BHQ1 dual-labeled oligonucleotides, (**B**) DNA duplexes formed by ROX-labeled or ROX-BHQ1 dual-labeled oligonucleotides and 22-nt partially complementary strand in 10 mM Tris–HCl buffer (pH 8.0) containing 0.1 mM EDTA and 100 mM KCl.

Annotations to Figure S2 and Table S4

DNA single-stranded fluorescent probes (Figure 7A). First, we compared the fluorescence intensities of ROX-labeled single-stranded DNAs lacking the BHQ1 quencher: 41G4-ROX, 41(GT)$_8$-ROX and 41T-ROX, and showed that G4 incorporation yielded a 1.6-fold decrease in the fluorescence intensity compared to that for DNAs carrying (GT)$_8$ or (T)$_{16}$ inserts (Figure S2A; Table S4). Therefore, the G4 structure itself may be a factor contributing to ROX fluorescence quenching. The ROX quenching due to the proximity of ROX and BHQ1 emerging upon G4 formation(Figure 7A) was observed by the comparison of fluorescence intensities of 41G4-ROX/41G4-ROX-BHQ1 and 41(GT)$_8$-ROX/41(GT)$_8$-ROX-BHQ1, the components of which lack or contain the BHQ1 quencher. The G4-mediated fluorescence decrease in the 41G4-ROX/41G4-ROX-BHQ1 pair (10.4 times) was greater than in 41(GT)$_8$-ROX/41(GT)$_8$-ROX-BHQ1 pair containing unstructured (GT)$_8$ loop (7.4 times) (Figure S2A; Table S4).

DNA duplexes with fluorescent labels (Figure 7B). According to our data, DNA duplex models labeled by ROX but lacking BHQ1 quencher (41G4-ROX/22, 41(GT)$_8$-ROX/22 and 41T-ROX/22) demonstrate almost identical and significantly reduced fluorescence intensities as compared to

corresponding ROX-labeled oligonucleotides (41G4-ROX, 41(GT)$_8$-ROX and 41T-ROX) under the same conditions (Figure S2B; Table S4). This is consistent with the large quenching effect of DNA strand hybridization on ROX fluorescence reported earlier [Nazarenko, I.; Pires, R.; Lowe, B.; Obaidy, M.; Rashtchian, A. Effect of primary and secondary structure of oligodeoxyribonucleotides on the fluorescent properties of conjugated dyes. *Nucleic Acids Res.* **2002**, *30*, 2089–2095]. Importantly, our data do not allow the G4 determination in a DNA duplex context. The difference in ROX fluorescence values is not observed even in dual-labeled DNA models 41G4-ROX-BHQ1/22 and 41(GT)$_8$-ROX-BHQ1/22, because the proximity of the ROX fluorophore and the BHQ1 quencher, provided by the DNA duplex formation, does not depend on the conformational state of the G4 motif or (GT)$_8$ repeat (Figure S2B; Table S4). Thus, ROX-labeled as well as ROX-BHQ1 dual-labeled DNA probes in 41/22 models turned out to be promising for detecting DNA duplex formation rather than G4 folding.

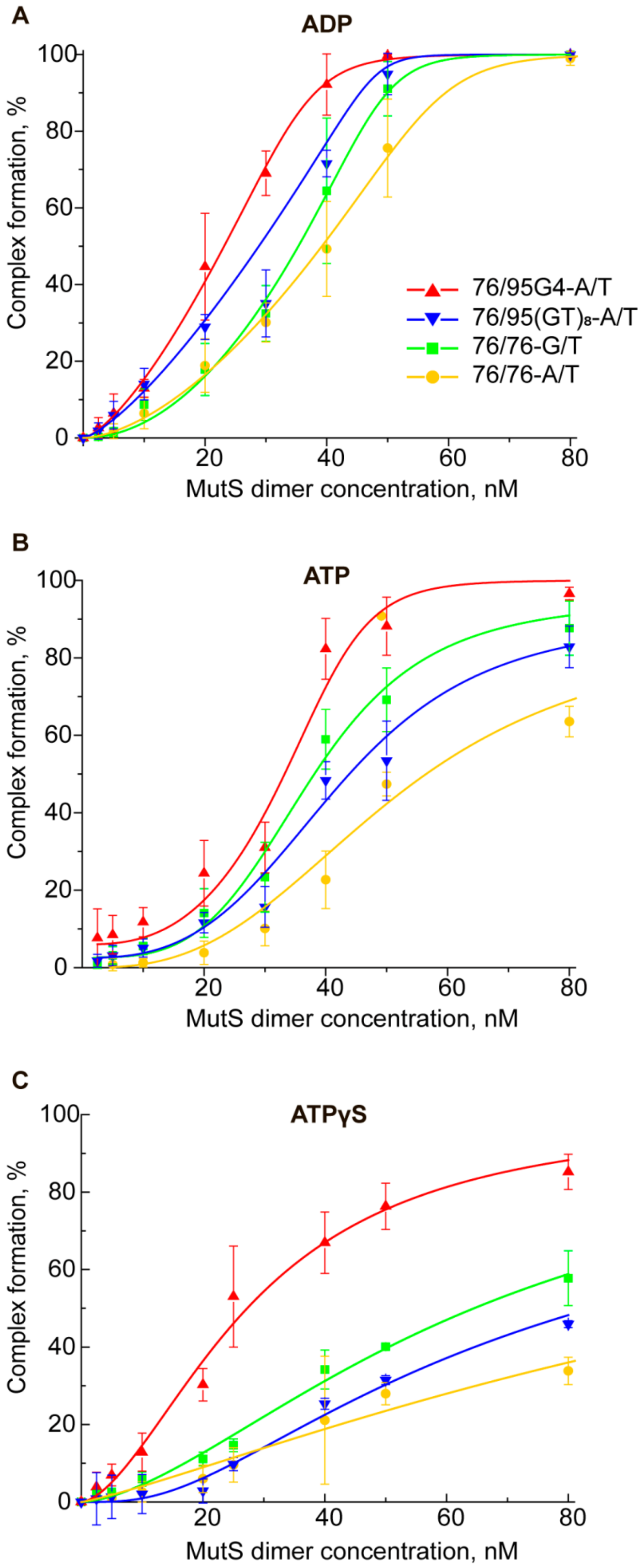

Figure S3. Binding of ecMutS to DNA ligands: 76/95G4-A/T, 76/95(GT)8-A/T, 76/76-A/T and 76/76-G/T, in the presence of nucleotide cofactors. (A) ADP, (B) ATP, (C) ATPγS ($p < 0.05$).

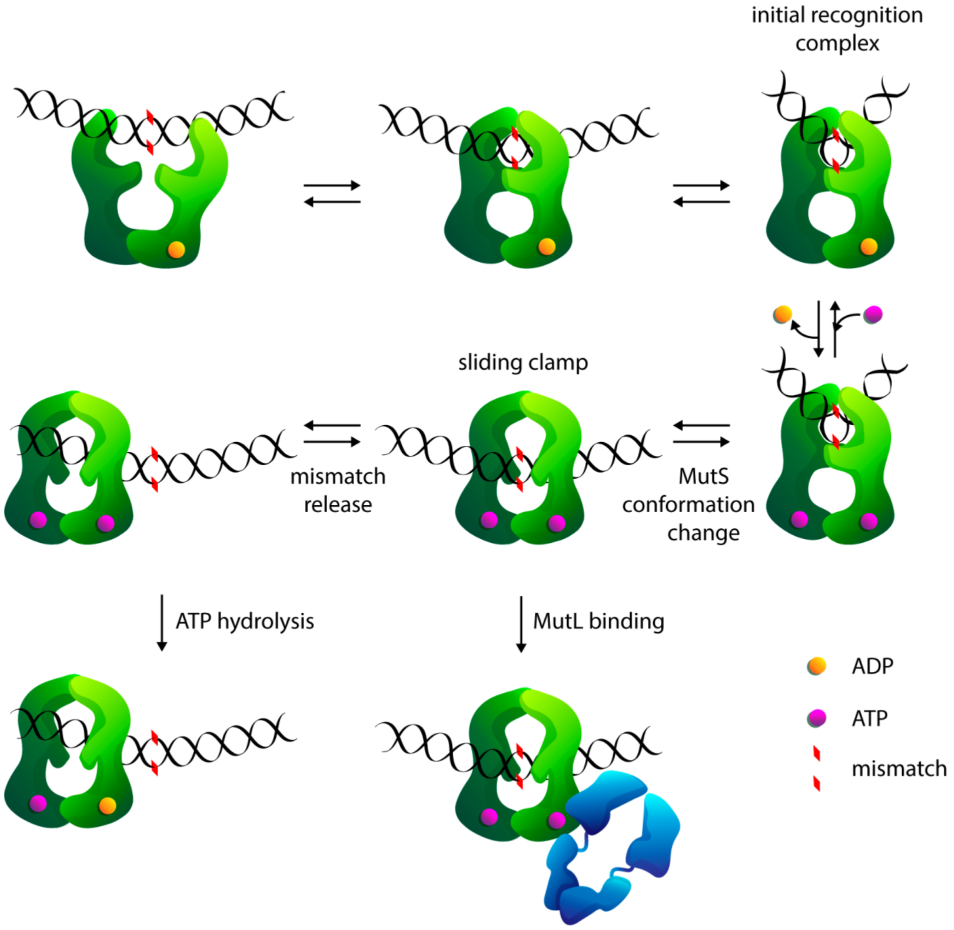

Figure S4. Scheme of conformational rearrangements of the MutS homodimer caused by its interaction with the DNA ligand and nucleotide cofactor (ADP and ATP) followed by hydrolysis of the cofactor and MutL binding according to [42].

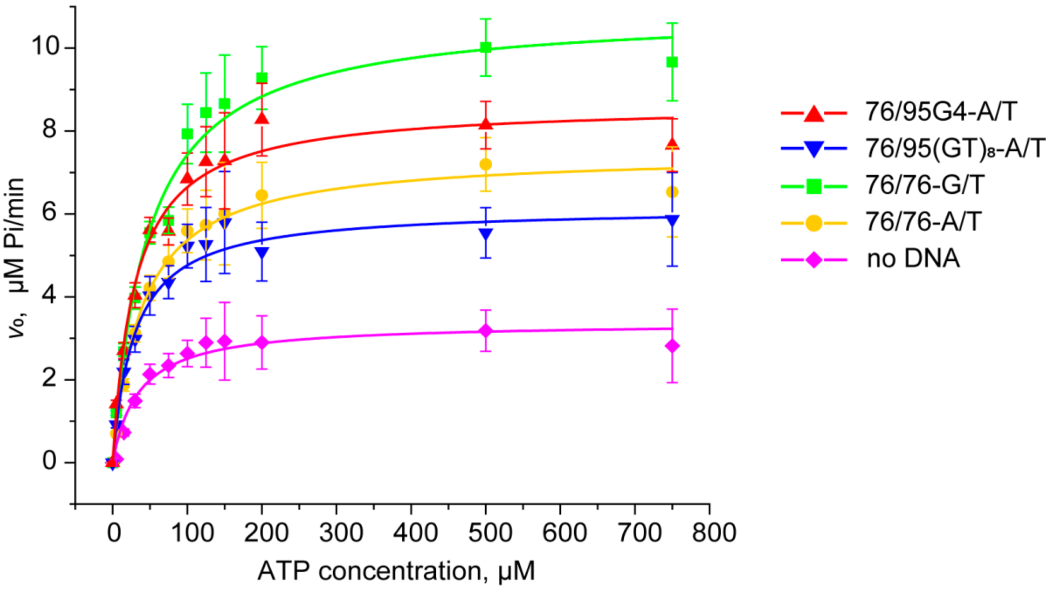

Figure S5. Dependence of initial rate of ATP hydrolysis reaction under MutS action in the absence and in the presence of DNA ligands (76/95G4-A/T, 76/95(GT)$_8$-A/T, 76/76-A/T and 76/76-G/T) ($p < 0.05$).

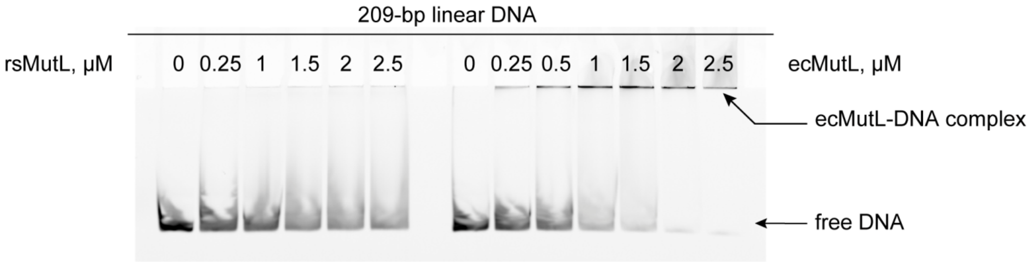

Figure S6. Analysis of complex formation between rsMutL (left panel) or ecMutL (right panel) (0–2.5 μM per dimer) and 100 nM fluorescent labeled 209-bp DNA visualized by mobility shift assay in 6% nondenaturing polyacrylamide gel.

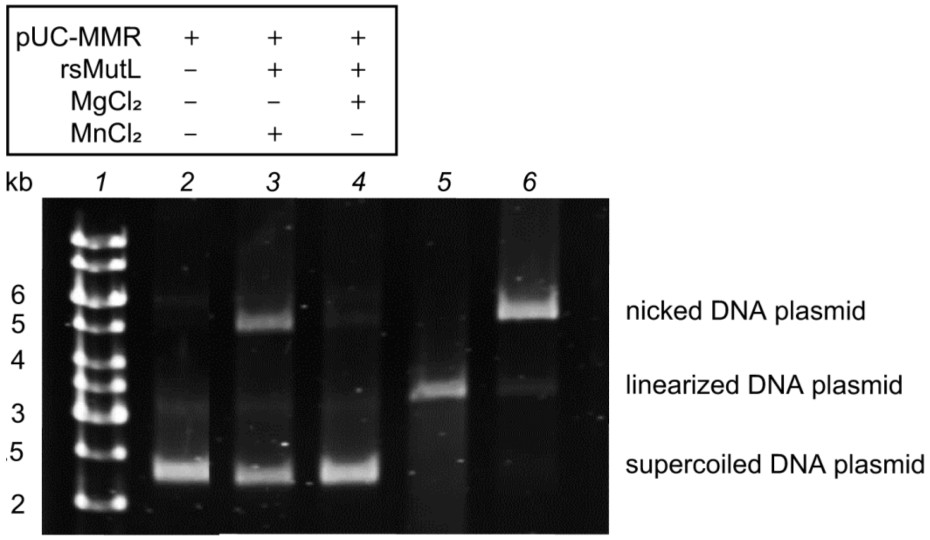

Figure S7. The hydrolysis of pUC-MMR plasmid by rsMutL at 37 °C for 60 min. The hydrolysis products were visualized in 1% agarose gel containing ethidium bromide. Lane *1* – DNA marker, lane *2* – control pUC-MMR plasmid, lane *3* – pUC-MMR hydrolysis by rsMutL in the presence of Mn^{2+}, lane *4* – pUC-MMR hydrolysis by rsMutL in the presence of Mg^{2+}, lane *5* – pUC-MMR hydrolysis with BamHI endonuclease, lane *6* – pUC-MMR cleavage with Bpu10I nicking endonuclease.

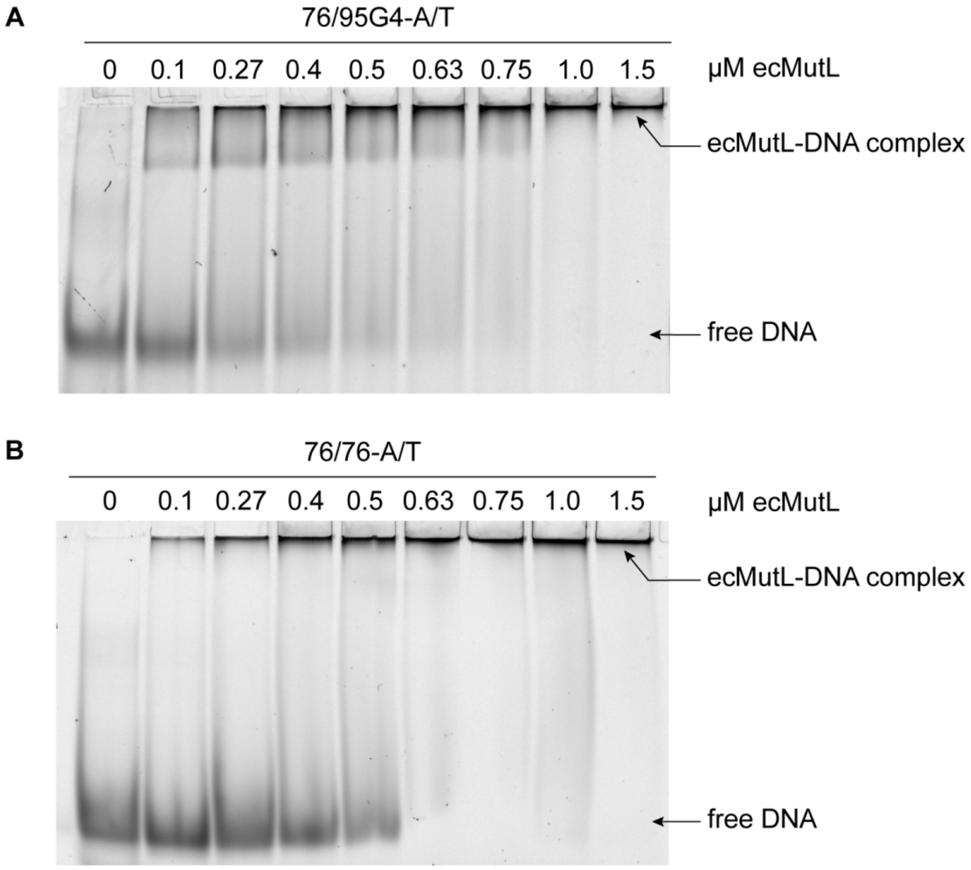

Figure S8. Analysis of complex formation between ecMutL (0–1.5 μM per dimer) and TAMRA-labeled DNA duplexes visualized by mobility shift assay in 6% nondenaturing polyacrylamide gel. (A) 100 nM 76/95G4-A/T; (B) 100 nM 76/76-A/T.
